# Posttranslational splicing modifications as a key mechanism in cytarabine resistance in acute myeloid leukemia

**DOI:** 10.1038/s41375-023-01963-4

**Published:** 2023-07-08

**Authors:** María Luz Morales, Roberto García-Vicente, Alba Rodríguez-García, Armando Reyes-Palomares, África Vincelle-Nieto, Noemí Álvarez, Alejandra Ortiz-Ruiz, Vanesa Garrido-García, Alicia Giménez, Gonzalo Carreño-Tarragona, Ricardo Sánchez, Rosa Ayala, Joaquín Martínez-López, María Linares

**Affiliations:** 1 Department of Translational Hematology, Instituto de Investigación Sanitaria Hospital 12 de Octubre (imas12), Hematological Malignancies Clinical Research Unit H12O-CNIO, Hospital 12 de Octubre – Centro Nacional de Investigaciones Oncológicas, CIBERONC, ES 28041 Madrid, Spain; 2 Department of Biochemistry and Molecular Biology, Veterinary School, Universidad Complutense de Madrid, ES 28040 Madrid, Spain; 3 Department of Medicine, Medicine School, Universidad Complutense de Madrid, ES 28040 Madrid, Spain; 4 Department of Biochemistry and Molecular Biology, Pharmacy School, Universidad Complutense de Madrid, ES 28040 Madrid, Spain

**Keywords:** Translational research, Acute myeloid leukaemia, Cancer, Cancer genetics

## Abstract

Despite the approval of several drugs for AML, cytarabine is still widely used as a therapeutic approach. However, 85% of patients show resistance and only 10% overcome the disease. Using RNA-seq and phosphoproteomics, we show that RNA splicing and serine-arginine-rich (SR) proteins phosphorylation were altered during cytarabine resistance. Moreover, phosphorylation of SR proteins at diagnosis were significantly lower in responder than non-responder patients, pointing to their utility to predict response. These changes correlated with altered transcriptomic profiles of SR protein target genes. Notably, splicing inhibitors were therapeutically effective in treating sensitive and resistant AML cells as monotherapy or combination with other approved drugs. H3B-8800 and venetoclax combination showed the best efficacy in vitro, demonstrating synergistic effects in patient samples and no toxicity in healthy hematopoietic progenitors. Our results establish that RNA splicing inhibition, alone or combined with venetoclax, could be useful for the treatment of newly diagnosed or relapsed/refractory AML.

## Introduction

There are now several targeted therapies approved for the treatment of acute myeloid leukemia (AML), including midostaurin, gilteritinib, CPX-351, enasidenib, ivosidenib, gemtuzumab ozogamicin, glasdegib and venetoclax [1–5]; however, their association with clinical benefit and longer survival times remains poor. Intensive chemotherapy with 7 days of cytarabine and 3 days of an anthracycline (commonly referred to as “7 + 3” regimens) together with transplantation in younger patients, or the use of hypomethylants in older patients, remains the standard of care for AML [6]. On standard chemotherapy, long-term survival of patients with AML is achieved in 35–45% of those younger than 60 years of age and in only 10–15% of those aged 60 and above. The outlook is particularly bleak for patients with drug resistance, as long-term survival is typically no higher than 10% [7]. Drug resistance includes both patients who do not respond initially, defined as primary refractory disease, and those who relapse after an initial response. Within this framework, relapse/refractory disease is the most common cause of death [7].

The presence of recurrent mutations in the spliceosome machinery, as well as aberrant splicing events, have been described as common alterations in AML disease [8, 9], and patients with AML who carry mutations in splicing proteins are characterized by a higher incidence of chemoresistance [10]. Moreover, mutations in spliceosome complex genes have been associated with the cause or consequence of drug resistance in AML, suggesting that targeting RNA splicing processes might be a novel approach to overcome treatment resistance [11]. The spliceosome is a macromolecular machine involving five small nuclear ribonucleoprotein particles (U1, U2, U4, U5, and U6 snRNPs) that recognize conserved nucleotide sequences across exon-intron junctions [12]. About 200 proteins have been identified as splicing factors, and most are members of the serine–arginine-rich (SR) and heterogeneous nuclear ribonucleoprotein (hnRNP) families [13]. The dysregulation of splicing factors may directly or indirectly affect many cellular processes in addition to RNA splicing [14]. The mechanisms controlling spliceosome activity and regulation include the posttranslational modification of spliceosomal proteins, which impacts their activity, subcellular localization, and proteasomal degradation [12]. For example, SR protein phosphorylation is necessary for the assembly of spliceosomal components, whereas dephosphorylation is essential for splicing catalysis [15].

Because functional changes in splicing regulatory proteins can promote oncogenesis through overexpression, alteration-of-function, and mutations, therapeutic targeting of the spliceosome holds promise as a novel cancer therapy [16]. Spliceosome inhibitors encompass a variety of small compounds that can prevent different steps of the splicing reaction. A number of splicing inhibitors interrupt the earliest stage of spliceosome assembly, such that no splicing complex is formed–for example, arginine N-methyltransferase 5 (PRMT5) inhibitors [17]. Other splicing inhibitors stall spliceosome assembly at the A complex, including madrasin and compounds targeting SF3B1 such as H3B-8800, or target kinases that regulate splicing factor activity and subcellular localization, including ATP-competitive inhibitors of the SR-phosphorylating kinases SRPK1/SRPK2 and CDC-like kinase inhibitors such as SPHINX31 and SRPKIN-1 [17]. Several clinical trials are investigating the therapeutic potential of these splicing inhibitors in different malignancies [13], including myelodysplastic syndromes, AML and chronic myelomonocytic leukemia (NCT02841540 and NCT03614728), non-Hodgkin lymphoma (NCT03666988 and NCT02783300), and advanced/metastatic solid tumors (NCT03854227) [16].

In the present study, we show that phosphorylation patterns of SR proteins are altered during the development of cytarabine resistance in AML, and that the combination of spliceosome inhibitors and other approved drugs, including the BCL2 inhibitor venetoclax, improves the therapeutic response in cells from patients with AML, even in a background of cytarabine resistance.

## Methods

### Cell culture, patients and healthy donors, and drugs

Human OCI-AML3, SKM-1 and THP-1 cell lines were cultured in RPMI-1460 (ref. BE12-702F/U1, Lonza, Walkersville, MD) supplemented with fetal bovine serum (ref. SV30160.03, Cytiva, Marlborough, MA) and antibiotics (ref. DE17-602E, Lonza). OCI-AML3 cytarabine-resistant cells (OCI-AML3_R) were generated from parental OCI-AML3 cells after sustained and cumulative exposure to 20 µM cytarabine. Samples from patients with AML (*n* = 75, median age = 60), myeloproliferative neoplasms (MPN, *n* = 12, median age = 75) and myelodysplastic syndromes (MDS, *n* = 11, median age = 78) and from 18 age-matched healthy donors from the same population-based studies were employed for analysis. Demographic and clinical features of the subjects are reported in Supplementary Table S1. The study was approved by the *Comité Ético de Investigación Clínica* of the *Instituto de Investigación Biomédica* of the *Hospital 12 de Octubre*, and all patients and donors provided written informed consent in accordance with the Declaration of Helsinki. For patients drug response categorization, we rely on ELN recommendations [6]: Non-responders or refractory (lack of substantial response or increase in the percentage of blasts in the bone marrow after treatment); Responders (<5% bone marrow blast); Relapse (reappearance of ≥5% bone marrow blasts). In addition, it was verified that the patients drug response categorization was not influenced by age. All drugs were purchased from Selleck Chemicals (Houston, TX), MedChemExpress (Monmouth, NJ), or were supplied by Vivia Biotech (Madrid, Spain) and the Pharmacy Department of the *Hospital 12 de Octubre*. For details see Supplementary Material.

### Analyses of public databases

We used the Gene Expression Profiling Interactive Analysis (GEPIA2) web-server [18] to compare gene expression profiles between patients with AML from The Cancer Genome Atlas (TCGA)-LAML project (*n* = 173) [19] and healthy controls from the GTEx project (*n* = 70) [20]. For details see Supplementary Material.

### RNA and DNA analysis

Gene expression levels of *SRRM2*, *SRSF12* (Unique Assay ID: qHsaCED0046512 and qHsaCED0045641, respectively, Bio-Rad Laboratories, Hercules, CA) and β-glucuronidase (*GUS*) (ref. 4304970, ThermoFisher Scientific, Waltham, MA) were measured by qPCR and quantified using the comparative cycle threshold (2ΔCt) method [21].

RNA libraries of paired samples from 25 patients with AML at diagnosis and cytarabine-resistance moments were generated following the KAPA RNA HyperPrep kit with RiboErase (HMR) protocol (KR1351-v1.16; Kapa Biosystems, Wilmington, MA) and sequenced on the NextSeq 500/550 platform (Illumina, San Diego, CA). Differential gene expression (DGE) analysis was performed using DESeq2 [22]; gene clustering analysis was performed based on the partitioning around medoids (PAM) algorithm [23]; and gene ontology (GO) overrepresentation analysis was performed using the clusterProfiler R package [24].

Differential exons usage analysis was carried out by Dreamgenics S.L. (Oviedo, Spain) using the DEXseq package [25]. Variants in the DNA sequence were studied with a customized next-generation sequencing (NGS) myeloid panel of 32 genes frequently mutated in myeloid diseases, as described [26, 27]. For details and analysis descriptions see Supplementary Material.

### LC-MS/MS analysis

Phosphoproteomic studies of bone marrow mononuclear cells (BMMCs) from paired samples of patients with AML (*n* = 3) (diagnosis and resistance) were conducted following the standard filter-aid preparation method and processed by IMAC and liquid chromatography tandem-mass spectrometry (LC-MS/MS) as described [28]. Differential phosphoproteomics analysis was performed using the DEP v1.12.0 R package [29]. For details see Supplementary Material.

### Immunohistochemistry

Methanol-fixed bone marrow smears from patients with AML at diagnosis (*n* = 64) and at resistance (*n* = 4) and after cytarabine treatment (*n* = 3) (see Supplemental Table S1) were stained with anti-SR protein family (ref. MABE126) or anti-phosphoepitope SR protein (ref. MABE50; all from Sigma-Aldrich, Madrid, Spain) antibodies. Signals were detected with an anti-mouse horseradish peroxidase-conjugated secondary antibody (ref. #8125, Cell Signaling Technology, Danvers, MA). For details see Supplementary Material.

### Drug sensitivity assay

Growth analyses after monotherapy or combination treatments were performed in cells seeded at 3 × 10^4^ (cell lines) or 2 × 10^4^ (primary cells) in 96-microwell plates and exposed to different drug doses (Table 1). Cell viability was determined with Cell Counting Kit-8 reagent (ref. 96992, Sigma-Aldrich) after 48 or 72 h. The half maximal inhibitory concentration (IC_50_) values were determined by nonlinear regression using GraphPad Prism 5.01 (La Jolla, CA), and the combination index (CI) and normalized isobolograms were obtained using Compusyn software (Combosyn Inc., Paramus, NJ) [30]. For details see Supplementary Material.

**Table 1:** Dose ranges of the different drugs studied in monotherapy or combination for in vitro and ex vivo experiments.

Sample	Drug	Treatment doses (Range from - to)	
		Monotherapy	Combination for OCI-AML3 and OCI-AML3_R1
OCI-AML3 SKM-1 THP-1 OCI-AML3_R	Cytarabine	50 µM–282 pM For OCI-AML3_R1: 16.67 mM–94 nM	0.62 µM–22.8 nM for OCI-AML3_R1: 1.85 mM–68 μM
	H3B-8800	1 mM–0.01 pM	100 µM–10 pM
	Madrasin	50 µM–24.41 nM	-
	SPHINX31	500 µM–2.82 nM	-
	SRPKIN-1	500 µM–2.82 nM	-
	Glasdegib	500 µM–244 nM	125 μM–15.63 µM
	Midostaurin	25 µM–12.8 pM	2 µM–16 nM
	Venetoclax	1 mM–20.48 pM	10 µM–80 nM
	Azacitidine	25 mM–512 pM	1.6 µM–12.8 nM
	Decitabine	4 mM–82 pM	0.8 mM–6.4 µM
Ex vivo (AML patients)	H3B-8800	10 µM–0.1 fM	10 µM–0.1 fM
	Venetoclax	111.11 µM–14.85 nM	10 µM–80 nM

### Colony-forming unit assay

CD34+ cells from BMMCs of healthy donors were isolated with the MACs CD34 MicroBead Kit (ref. 130-046-703, Miltenyi Biotec S.L., Madrid, Spain) and cultured in methylcellulose medium (Methocult Express; ref. 4437, StemCell Technologies, Vancouver, Canada) with different drug doses. Colony-forming units (CFU-granulocyte-monocyte and erythroid colonies) were scored after 14 days. For details see Supplementary Material.

### Statistical analysis

Statistical analyses were performed with GraphPad Prism 5.01 software. Comparisons between two groups were performed using the parametric Student’s *t*-test or the non-parametric Mann-Whitney *U* test, for unrelated samples, and the Wilcoxon signed-rank test, for related samples. Differences were considered as statistically significant at *P* ≤ 0.05. Data are presented as the mean 土 standard error of the mean (SEM).

## Results

### Splicing related genes are altered in AML and its response to cytarabine treatment

**Fig. 1:**
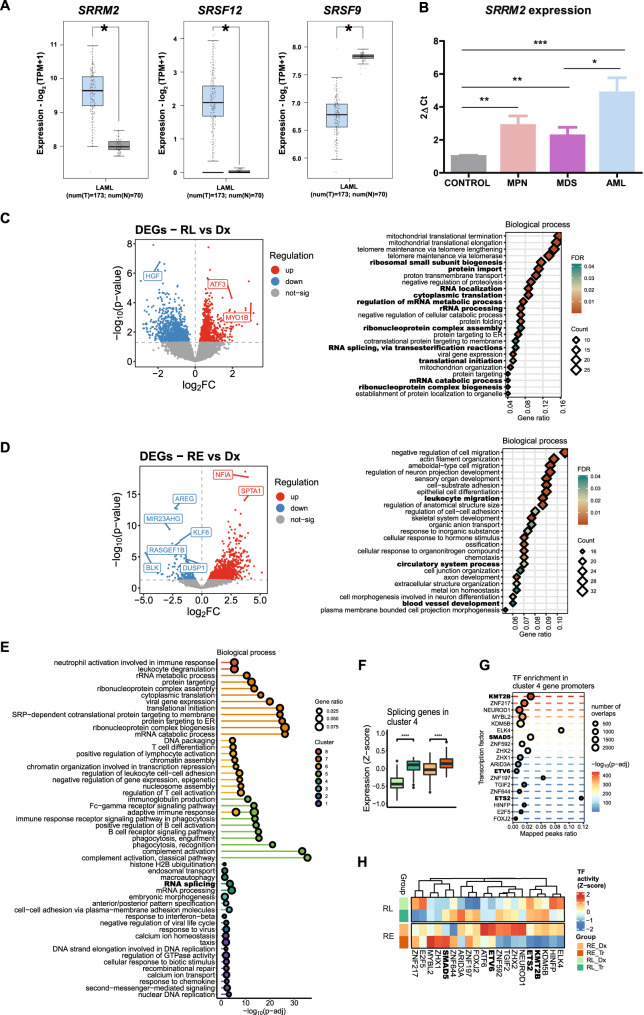
Splicing related genes are altered in AML and its response to cytarabine treatment. **A** GEPIA2-related gene expression comparison between patients in *The Cancer Genome Atlas – Acute Myeloid Leukemia* (TCGA-LAML; *n* = 173) and *The Genotype-Tissue Expression* bone marrow controls (GTEx; *n* = 70). **B** 2ΔCt values for *SRRM2* expression in bone marrow samples from patients with acute myeloid leukemia (AML; *n* = 16), myelodysplastic syndrome (MDS; *n* = 11) or myeloproliferative neoplasms (MPN; *n* = 12) and healthy controls (*n* = 15) at diagnosis, normalized to *GUS* expression. **C** Volcano plots showing differentially expressed genes (DEGs) analysis distribution based on Fold Change (*x*-axis) and significance (*y*-axis) and overrepresentation analysis of gene ontology (GO) terms in DEGs for relapse after response (RL) patient samples. **D** Volcano plots showing DEGs analysis distribution based on Fold Change (*x*-axis) and significance (*y*-axis) and overrepresentation analysis of GO terms in DEGs for refractoriness (RE) patient samples. **E** Overrepresentation analysis of GO terms in 8 gene clusters, where genes were clustered based on partitioning around medoids (PAM) algorithm, showing top 10 most significant terms in each cluster with 5% FDR. **F** Boxplot showing the gene expression in each group of genes found in the RNA splicing GO term in cluster 4. **G** Transcription factor (TF) enrichment analysis of TF binding sites in cluster 4 gene promoters, showing the 20 TFs represented in Fig. 1H. **H** Heatmap showing TF activity estimated from TF regulons expression representing top 20 most variable TFs across samples. Boxplot elements: center line, median; box limits, upper and lower quartiles; points, samples. Dx: Diagnosis, Tr: After cytarabine treatment. *P ≤ 0.05, **P ≤ 0.001, ***P ≤ 0.0001, ****P ≤ 0.00001.

Previously, it has been observed that 14% of AML patients present alterations in spliceosome-complex genes [19]. Now, by using public repository data we have showed significant differences in the mRNA expression levels of three genes encoding SR proteins splicing factors: *SRRM2*, *SRSF12*, and *SRSF9* (Fig. 1A). Expression levels of the two overexpressed genes (*SRRM2* and *SRSF12*) were studied in a cohort of 54 patients from *Hospital 12 de Octubre*. Results showed that serine–arginine repetitive matrix protein 2 (*SRRM2*) was significantly overexpressed in the myeloid diseases AML (mean = 4.85 ± 0.92; *P* = 0.0003), MPN (mean = 2.87 ± 0.58; *P* = 0.0015), and MDS (mean = 2.25 ± 0.51; *P* = 0.0094) when compared with controls, with expression being significantly higher in AML than in MDS (*P* = 0.0382) (Fig. 1B). We also detected higher levels of serine–arginine-rich splicing factor 12 (*SRSF12*) in myeloid diseases than in controls, although the differences were not significant (Supplementary Fig. S1A).

To evaluate the transcriptional alterations associated with treatment, we performed a DGE analysis from paired AML samples to contrast diagnosis and cytarabine-resistance moments, revealing transcriptomic changes in cytarabine-resistant groups (Fig. S2G). We spotted differences in expression levels of genes involved in the regulation of SR proteins or their targets both in relapse (*HGF*, *ATF3* and *MYO1B*) or refractory (*AREG*, *MIIR23AHG*, *KLF6*, *RASGEF1B*, *BLK*, *DUSP1*, *NFIA* and *SPTA1*) (Fig. 1C, D). Subsequent functional enrichment analysis confirmed an overrepresentation of GO terms related to the RNA functionality, such as the regulation of mRNA metabolic and catabolic process, RNA splicing and localization or ribonucleoprotein complex biogenesis and assembly (Fig. 1C). To identify specific gene signatures of treatment and type of response in patients, we performed a gene clustering analysis based on PAM algorithm (Supplementary Fig. S2E). Interestingly, gene signatures related to RNA splicing processes were overrepresented in cluster 4 (Fig. 1E) and significantly upregulated in relapse or refractory patients after cytarabine treatment (Fig. 1F). We conclude that the gene expression of SR proteins appears to be linked to resistance to first line treatment of AML.

In order to explore the regulatory drivers underlying these processes, we estimated transcription factor activities based on expression data (Fig. 1H). We found 4 transcription factors (*KMT2B*, *ETS2*, *SMAD5* and *ETV6*) among the 20 most variable in activity across samples (Fig. 1H), that have been linked to cytarabine resistance in previous studies [31–34]. Finally, we performed an enrichment analysis of transcription factor binding sites at promoters of genes harbored at cluster 4. Selecting those previously highlighted in the activity estimations (Fig. 1H), we observed that KMT2B showed the most significant enrichment, and ETS2 the highest ratio of overlapped regions (mapped peaks ratio) (Fig. 1G).

### Levels of phosphorylated SR proteins are related to cytarabine response

**Fig. 2:**
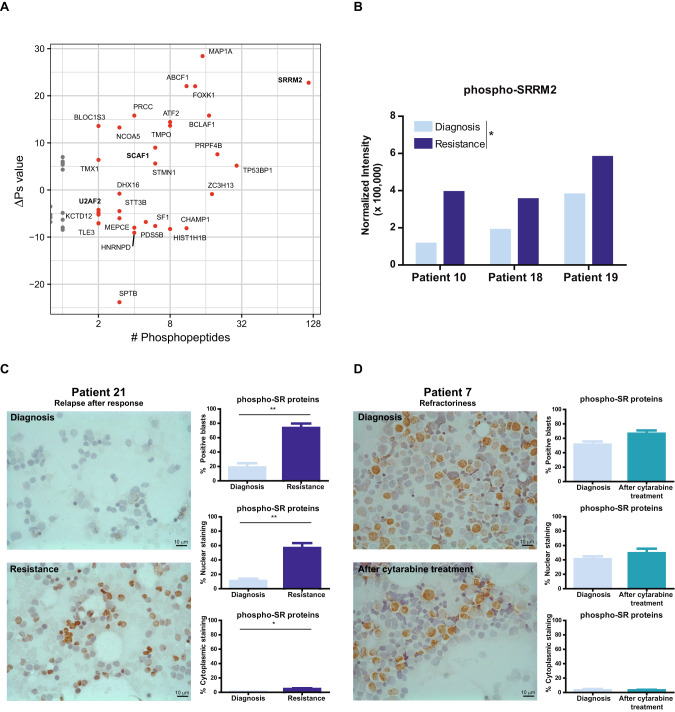
SR proteins phosphorylation is elevated in cytarabine resistant patients. **A** Paired phosphoproteomic profile analysis revealed significantly higher changes in some SR proteins (SRRM2, SCAF1 and U2AF2) phosphorylation between diagnosis and cytarabine-resistance, represented by the sum of phosphopeptide signal (sum of log_2_FC values, *y*-axis) according to the number of phosphopeptides (*x*-axis), with a *p*-value cutoff of 0.01. **B** Intensity of phosphoepitopes from SRRM2 was higher after treatment in the three patients with AML. **C** Immunohistochemistry and percentage of positive blasts, and nuclear and cytoplasmic staining of phospho-SR proteins in paired bone marrow smears at diagnosis and resistance in 4 patients with AML that responded to cytarabine and further relapsed. **D** Immunohistochemistry and percentage of positive blasts, and nuclear and cytoplasmic staining of phospho-SR proteins in paired bone marrow smears at diagnosis and after cytarabine treatment in 3 AML patients that did not initially respond. Remaining bone marrow smears samples analyzed are included in Supplementary Fig. S3A. Scale bar, 10 µm.

To question whether posttranslational modifications also were implicated in cytarabine resistance, we analyzed the phosphoproteomic profile of paired AML samples (diagnosis *vs* resistance) by LC-MS/MS. Analysis of the intensity of the phosphoepitopes between the two groups revealed significant differences in the phosphorylation of several SR proteins, with SRRM2 showing the greatest changes after treatment (Fig. 2A). Proteins whose phosphorylation significantly changed after treatment were selected and compared between patients. Results showed that SRRM2 phosphorylation was significantly higher in all patients with cytarabine resistance (diagnosis: 2.26 ± 0.79; resistance: 4.42 ± 0.70; *P* = 0.023; Fig. 2B).

We sought to validate these results by immunohistochemistry (IHC) of paired bone marrow smears from patients with AML (diagnosis *vs* resistance), finding significantly higher levels of phospho-SR proteins at resistance in cytarabine-responder patients that further relapsed (Fig. 2C and Supplementary Fig. S3A). Given the relationship between SR protein phosphorylation status, subcellular localization and functionality, we classified staining as nuclear or cytoplasmic. A significant increase in both was found in resistant samples. To test whether these differences could be explained by an increase in the basal levels of SR proteins, total, nuclear, and cytoplasmic staining were compared in paired samples, which revealed no evident differences (Supplementary Fig. S3B), suggesting a specific role for phospho-SR proteins in cytarabine resistance development.

To study whether phospho-SR proteins were also elevated in patients that did not initially respond to cytarabine treatment, we analyzed their levels in paired AML samples (diagnosis *vs* after cytarabine treatment). In these patients, the phospho-SR protein levels after treatment were similar to patients that relapse, however, they presented remarkable higher ones at diagnosis. (Fig. 2D and Supplementary Fig. S3A), indicating their potential utility as a biomarker of cytarabine response.

**Fig. 3:**
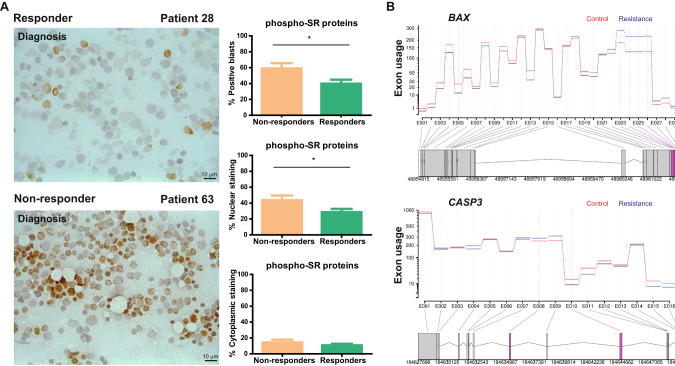
Phosphorylation of SR proteins could predict cytarabine response and are related to aberrant splicing process. **A** Immunohistochemistry and percentage of positive blasts, and nuclear and cytoplasmic staining of phospho-SR proteins in cytarabine non-responders and responders. Remaining bone marrow smear samples are included in Supplementary Fig. S4. **B** Transcriptional profile of SR protein target genes *BAX* and *CASP3* showing differential exons usage between samples at diagnosis and resistance (*n* = 25). Transcriptional profiles of *H2AFY*, *DPPIII*, *WAC*, *DEPDC5*, *Ki-67*, *MYLK*, and *S6K1* are included in Supplementary Fig. S5B. Adjusted *p*-value ≤ 0.05. Scale bar, 10 µm.

We thus examined whether phospho-SR protein levels could serve as a predictor of cytarabine response at the time of diagnosis in a cohort of 64 patients with AML with differential responses to cytarabine (43 responded to therapy [responders] and 21 were refractory [non-responders]). Results showed that non-responders had significantly higher levels of phospho-SR protein staining in positive blasts than responders (non-responders: 59.03 ± 6.61%; responders: 39.87 ± 4.95%; *P* = 0.018), with predominantly nuclear staining (Fig. 3A and Supplementary Fig. S4).

To discard the possibility that these differences could be explained by the patients’ molecular backgrounds, we compared their mutational profiles in different functional categories, including splicing genes, and found no differences (Supplementary Fig. S5A). As phosphorylation of SR proteins could affect their role in splicing, favoring the selection of different transcripts, we used our RNA-seq data to compare the transcriptional profile of several described target genes of SR proteins [35–43] in a cohort of 25 patients with AML (diagnosis *vs* cytarabine resistance). Profiling analysis identified differential exon usage between the two groups (Fig. 3B and Supplementary Fig. S5B), and revealed that alterations in SR protein phosphorylation correlated with differences in their activity in alternative splicing processes for several SR protein targets, including the SRRM2 pro-apoptotic targets *BAX* and *CASP3*; the targets *MYLK* or *Ki-67*; the U2AF1 target *H2AFY* and *WAC*; and the SRSF1 target *S6K1* [36–38].

### Splicing inhibitors alone or in combination with frontline AML protocols are effective for AML treatment

**Fig. 4:**
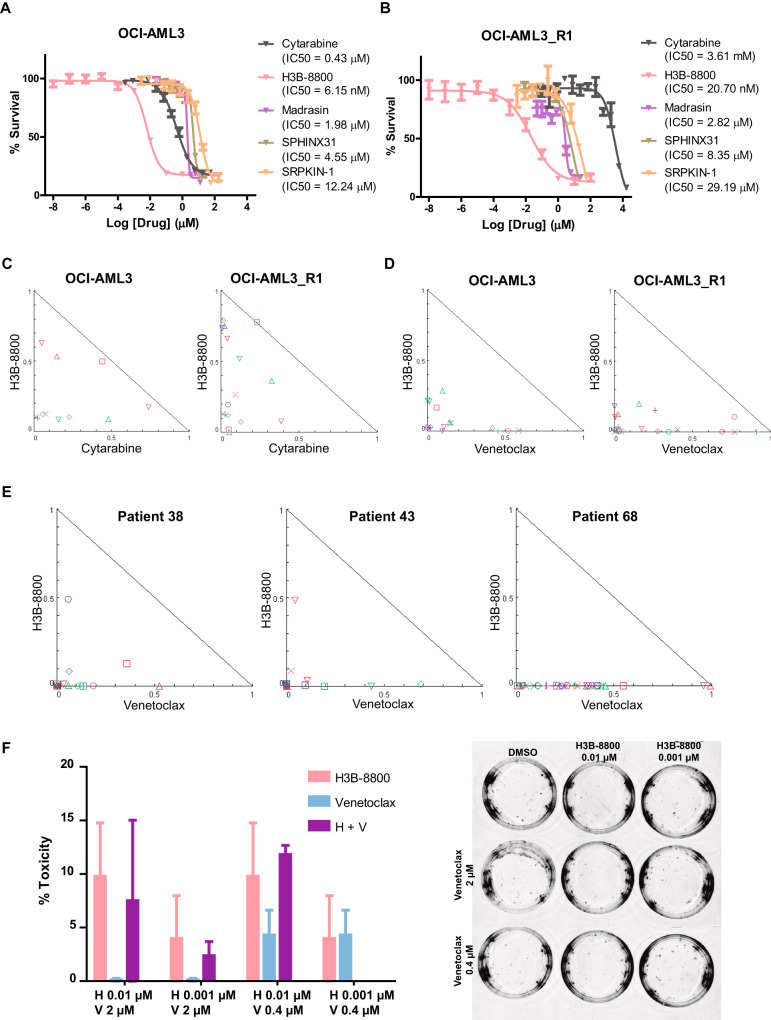
The combination of H3B-8800 plus venetoclax shows synergistic effects in leukemic cells and non-cytotoxicity in healthy donor hematopoietic precursors. **A** Dose-response curves of OCI-AML3 cells for cytarabine and the splicing inhibitors (H3B-8800, madrasin, SPHINX31 and SRPKIN-1). **B** Dose-response curves of cytarabine-resistant clone OCI-AML3_R1 for cytarabine and the splicing inhibitors. **C** Normalized isobolograms for H3B-8800 in combination with cytarabine in OCI-AML3 and OCI-AML3_R1 cell lines. **D** Normalized isobolograms for H3B-8800 in combination with venetoclax in OCI-AML3 and OCI-AML3_R1 cell lines. **E** Normalized isobolograms for H3B-8800 plus venetoclax combination in bone marrow mononuclear cells (BMMC) from 3 patients with AML at diagnosis. **F** Percentage of toxicity associated with H3B-8800, venetoclax and their combination in CD34 + BMMC from 3 healthy donors.

To evaluate whether splicing inhibition could be a potential new target in AML or could overcome cytarabine resistance, we tested several inhibitors in vitro using three different AML models of subclinical disease and stage (OCI-AML3, SKM-1 and THP-1) with different sensitivity to cytarabine. Results showed that several spliceosome inhibitors inhibited cell growth in vitro in the micromolar range, including madrasin, SPHINX31 and SRPKIN-1; notably, H3B-8800 inhibited cell growth in the low nanomolar range (Fig. 4A and Supplementary Fig. S6A).

As OCI-AML3 cells were the most sensitive to cytarabine, we used this cell line to generate a cytarabine-resistant model by continuous exposure to cytarabine up to 20 µM (OCI-AML3_R). No differences in the mutational status of most frequent genes mutated in AML [26, 27] were found between parental OCI-AML3 cells and OCI-AML3_R cells (*n* = 3). We then used a limiting dilution protocol and isolated 20 independent cytarabine-resistant clones (Supplementary Fig. S7A). Of note, 30% of the cytarabine-resistant clones exhibited increased levels of phospho-SR proteins with respect to parental OCI-AML3 cells (Supplementary Fig. S7B). Analysis of the cell clones showing the greatest staining (OCI-AML3_R1: 34.6, R2: 17.4 and R16: 2.1 times of increase in staining normalized to parental cells) revealed that clones maintained cytarabine resistance and that response to spliceosome inhibitors were similar between sensitive and resistant OCI-AML3 cells (Fig. 4B and Supplementary Fig. S6B).

The combination of cytarabine plus H3B-8800 was synergic in the sensitive but also in the resistant clone selected for further analysis (OCI-AML3 and OCI-AML3_R1 respectively) (Fig. 4C), supporting their potential as a therapeutic option even in a background of cytarabine resistance. However, because cytarabine administration in patients with AML results in bone marrow toxicity [44], we tested the combination of H3B-8800 with other clinically approved therapies for AML, including azacitidine, decitabine, glasdegib, midostaurin and venetoclax, to identify the most appropriate and effective combination. We calculated the IC_50_ values of each drug in OCI-AML3 and OCI-AML3_R1 cells (Supplementary Fig. S8), finding that none showed cross-resistance with cytarabine and had similar IC_50_ values in both cell lines, demonstrating their utility in the context of cytarabine resistance. Evaluation of the combination of H3B-8800 with each drug (Table 1) revealed synergic effects in all cases (Fig. 4D and Supplementary Fig. S9), with the combination of H3B-8800 and the BCL2 inhibitor venetoclax being the most potent (with most of the CI values ≤ 0.5) in both OCI-AML3 and OCI-AML3_R1 cell lines.

### H3B-8800 plus venetoclax shows a synergistic and non-cytotoxic profile in ex vivo AML and control samples

Similar to the results in cell lines, the combination of H3B-8800 and venetoclax showed enhanced antileukemic activity in ex vivo BMMCs isolated from three patients with AML (Fig. 4E). Strong synergic effects of the combination (CI ≤ 0.5) were observed in each case, in line with the effects observed in vitro. Finally, to test whether this combination could affect the colony formation of granulocyte-monocyte or erythroid colonies, we tested different combinational doses in healthy CD34+ donor cells. The combinations tested (H3B-8800 0.01 µM and 0.001 µM with venetoclax 2 µM and 4 µM) showed lower toxicity than the sum of the monotherapy doses and, in all cases, less than 15% (Fig. 4F). Therefore, the same combination doses that inhibited leukemic cell growth (toxicity percentage: 71.32, 21.11, 78.04 and 17.47), had no effect on the proliferation of healthy hematopoietic progenitor cells (7.51, 2.38, 11.82, and 0 respectively).

## Discussion

Aberrant mRNA splicing in AML has been previously demonstrated [9], but its potential role in drug resistance was unclear. Here we demonstrate that posttranslational modifications of splicing factors are involved in cytarabine resistance and can be effectively and safely targeted in AML. Cytarabine remains the most effective single-agent treatment for AML [45]; yet, while 70–80% of patients achieve remission following induction chemotherapy, 80% of these patients ultimately relapse and no salvage regimen is currently available for treatment failure [46]. Accordingly, overriding cytarabine resistance remains an unmet clinical need.

Large scale studies such as those of TCGA, which have analyzed the genome of 200 adult patients with de novo AML [19], have attempted to distinguish the main genetic alterations implicated in the pathogenesis of the disease. Using these data, here we verified that the expression of the genes encoding the SR proteins SRRM2, SRSF12, and SRSF9 differ between AML and control samples. Genetic mutations [10, 11, 19], differences in expression [36, 47–50] or functional failure [9] of spliceosome machinery components, such as SR proteins, have been previously described, providing a rationale for the exploration of new treatments for disease. We confirm the overexpression of *SRRM2* in a series of patients with myeloid disease, with a particularly significant increase in expression in AML. These findings point to a possible role for gene overexpression in the development of these diseases. *SRRM2* overexpression has been observed in nasopharyngeal carcinoma, and its silencing in cellular models of the disease diminishes proliferation, blocks the cell cycle, and enhances apoptosis [36].

Mutations in mRNA splicing genes has been linked to drug resistance development in AML [8, 11, 51]. Now, we have compared the transcriptional profile between the diagnosis and resistance moments in AML patients. We have found differentially expressed genes that have been previously linked to the functionality of SR proteins through different mechanisms [52–59]. For example, *HGF* [53], *MIR12AHG* [54], *BLK* [55], *KLF6* [56] and *RASGEF1B* [57] appear to alter different SR proteins or RNA-binding proteins; while others, such as *MYO1B* [58] or *SPTA1* [59] can be targets of splicing factors. Furthermore, the GO terms analysis is consistent with the working hypothesis and previous results, pointing to transcriptional changes related to the regulation of RNA splicing processes [11]. Interestingly, splicing instances appeared as one of the most overrepresented processes in gene signatures clustered by cytarabine response. Additionally, we have also found changes in activity and enrichment of some transcription factors that could be involved in the regulation of these processes. These results provide novel insights into the regulatory mechanisms underlying this process, since some of them have been previously described as mediators of cytarabine resistance. That is the case of *KMT2B* [31], *ETS2* [32], *SMAD5* [33] and *ETV6* [34]. For instance, *ETV6*, involved in different types of leukemia [60] is downregulated in cytarabine-resistant tissues [34], and *ETV6* knockout cell lines exhibit an increased gene expression of splicing factors [61]. Therefore, the estimated lower activity of ETV6 in cytarabine-resistant samples (Fig. 1H) could explain the robust upregulation of splicing factor genes observed in Fig. 1F.

We show by phosphoproteomics and immunohistochemistry that the phosphorylation of SR proteins increases after the development of acquired resistance to cytarabine. This increase was not related to changes in the location or retention of SR proteins, but rather to changes in their activity/functionality [15, 62, 63]. Based on these results, we postulate that chemotherapy enriches for cells that present with altered phosphorylation at diagnosis, which survive during treatment and become the major cell population at the time of drug resistance. To date, only a few studies have analyzed the complete proteomic profile of AML using ex vivo samples [64–68]. For example, Aasebø et al. [67] analyzed the proteomic profile of patients with AML with different responses to cytarabine at diagnosis, and identified alterations in RNA processing in relapsing patients. Their results support our findings showing that variations in RNA processing are related to cytarabine resistance. Moreover, we found that patients with primary resistance (non-responders) had higher levels of phospho-SR proteins at diagnosis than those who achieved complete remission (responders), consistent with the behavior observed in our findings in gene expression changes and transcription factor dynamics. Our validation in a cohort of 64 patients with AML proves the usefulness of quantifying phospho-SR protein levels as a predictive biomarker of cytarabine response not linked to the presence of specific mutations in the components of the splicing machinery. This may allow better stratification of patients to identify those non-responders that might benefit from therapies less aggressive than cytarabine, which has high hematological, neurological, hepatic and gastrointestinal toxicity [44].

Splicing alterations in cancer can modify the functionality of oncogenes, tumor suppressor proteins, splicing factors, apoptosis proteins, and also cell proliferation [11, 12, 14]. Notably, in patients with AML, malfunction of these process has been linked to alterations in the splicing of several relevant signaling pathways such as FLT3, CD13, cKIT, NOTCH, PI3K or MAPK, which are involved in the regulation of the cell cycle, apoptosis, cellular transformation and splicing [9]. To confirm that the splicing function of SR protein is altered in AML, we tested several SR protein targets, finding a pattern of differential exon usage between the time of diagnosis and drug resistance in paired samples from 25 patients with AML. These modifications thus lead to splicing failure, possibly favoring the generation of resistant clones. Accordingly, splicing inhibition is of substantial interest as a therapeutic target. Pharmacological inhibition of splicing mainly affects cancer cells [16] (which show splicing deficiencies and do not have sufficient canonical mRNA to survive), while healthy cells can tolerate a certain degree of inhibition because they still have sufficient canonical splicing products [37].

Preclinical studies with the splicing inhibitor H3B-8800 have revealed its potential in the treatment of myeloid diseases that carry mutations in splicing factors [16, 37, 69]. Our results reveal that it might be equally useful for treating AML, as shown in our cell models that have no mutations in splicing genes, as has been previously suggested [70]. However, as with most drugs used in the treatment of AML, clinical trial results of H3B-8800 published to date show that although the treatment is safe for patients [37], it is not sufficient to induce a complete response [69, 71]. We tested different combinations of H3B-8800 in vitro with other inhibitors that are approved for AML treatment, finding that all combinations were synergistic. The combination of H3B-8800 with venetoclax exhibited the most potent drug synergy in vitro in both cytarabine-sensitive and -resistant cells. These synergic effects are supported by recent literature demonstrating that a combination based on splicing and BCL2 inhibition is effective in other hematological malignancies, including multiple myeloma [72] and chronic lymphocytic leukemia [73]. Indeed, the use of venetoclax in AML inhibits the protective mechanisms characteristic of stem cells that defend them from the pharmacological action of several drugs [74]. Furthermore, it has been reported that *SRRM2* silencing increases the levels of pro-apoptotic proteins such as Bax or caspase 3, while decreasing the levels of anti-apoptotic proteins such as BCL2 or Ki-67 [36]. It could therefore be expected that the high expression of SRRM2 reported in AML would favor an increase in BCL2 expression, although this needs further research. Finally, we evaluated the efficacy of the combination in primary AML cells from patients at diagnosis, finding that the combination showed potent synergy in an ex vivo context, which might allow a reduction in the dosage of each drug to achieve the same effects. Reassuringly, hematotoxicity studies in progenitor cells from healthy donors revealed no changes in the formation of granulocyte-macrophage progenitor colonies or erythroid populations. This is especially relevant given that venetoclax administration has been halted in some patients with AML because of hematotoxicity [75], and so having another drug that acts synergistically with venetoclax could lower its dosage. Accordingly, combination of H3B-8800 and venetoclax might be an effective and safe treatment strategy for AML, but further research is required to understand the mechanism underlying their effects.

In sum, we demonstrate that altered phosphorylation of SR proteins is related to primary or secondary resistance to cytarabine, and might be useful to predict response. Moreover, inhibition of the splicing mechanism, alone or in combination with venetoclax, could be a good strategy for the treatment of newly diagnosed or relapse/refractory AML.

## Supplementary information

Supplemental Material (Leukemia - ProvAccepted)

## Data Availability

The RNA sequencing data generated and analysed during the current study are openly available in the BioProject repository (NCBI) with accession number PRJNA799381. The mass spectrometry proteomics data have been deposited to the ProteomeXchange Consortium via the PRIDE [76] partner repository with the dataset identifier PXD034010.

## References

[1] Burnett A, Stone R (2020). AML: new drugs but new challenges. Clin Lymphoma Myeloma Leuk.

[2] Kucukyurt S, Eskazan AE (2019). New drugs approved for acute myeloid leukaemia in 2018. Br J Clin Pharm.

[3] McMahon CM, Perl AE (2019). Management of primary refractory acute myeloid leukemia in the era of targeted therapies. Leuk Lymphoma.

[4] Saleh K, Khalifeh-Saleh N, Kourie HR (2020). Acute myeloid leukemia transformed to a targetable disease. Future Oncol.

[5] Daver N, Wei AH, Pollyea DA, Fathi AT, Vyas P, DiNardo CD (2020). New directions for emerging therapies in acute myeloid leukemia: the next chapter. Blood Cancer J.

[6] Döhner H, Estey E, Grimwade D, Amadori S, Appelbaum FR, Büchner T (2017). Diagnosis and management of AML in adults: 2017 ELN recommendations from an international expert panel. Blood.

[7] Short NJ, Rytting ME, Cortes JE (2018). Acute myeloid leukaemia. Lancet.

[8] de Necochea-Campion R, Shouse GP, Zhou Q, Mirshahidi S, Chen C-S (2016). Aberrant splicing and drug resistance in AML. J Hematol Oncol.

[9] Adamia S, Haibe-Kains B, Pilarski PM, Bar-Natan M, Pevzner S, Avet-Loiseau H (2014). A genome-wide aberrant RNA splicing in patients with acute myeloid leukemia identifies novel potential disease markers and therapeutic targets. Clin Cancer Res.

[10] Lindsley RC, Mar BG, Mazzola E, Grauman PV, Shareef S, Allen SL (2015). Acute myeloid leukemia ontogeny is defined by distinct somatic mutations. Blood.

[11] Zhou J, Chng W-J (2017). Aberrant RNA splicing and mutations in spliceosome complex in acute myeloid leukemia. Stem Cell Investig.

[12] Gonçalves V, Pereira JFS, Jordan P. Signaling pathways driving aberrant splicing in cancer cells. Genes (Basel). 2017;9. 10.3390/genes9010009. 10.3390/genes9010009 PMC5793162 29286307

[13] Lee SC-W, Abdel-Wahab O (2016). Therapeutic targeting of splicing in cancer. Nat Med.

[14] Dvinge H, Kim E, Abdel-Wahab O, Bradley RK (2016). RNA splicing factors as oncoproteins and tumour suppressors. Nat Rev Cancer.

[15] Zhou Z, Fu X-D (2013). Regulation of splicing by SR proteins and SR protein-specific kinases. Chromosoma.

[16] Bonnal SC, López-Oreja I, Valcárcel J (2020). Roles and mechanisms of alternative splicing in cancer - implications for care. Nat Rev Clin Oncol.

[17] Eymin B (2021). Targeting the spliceosome machinery: a new therapeutic axis in cancer?. Biochem Pharm.

[18] Tang Z, Li C, Kang B, Gao G, Li C, Zhang Z (2017). GEPIA: a web server for cancer and normal gene expression profiling and interactive analyses. Nucleic Acids Res.

[19] Ley TJ, Miller C, Ding L, Raphael BJ, Mungall AJ, Robertson AG (2013). Genomic and epigenomic landscapes of adult de novo acute myeloid leukemia. N. Engl J Med.

[20] Lonsdale J, Thomas J, Salvatore M, Phillips R, Lo E, Shad S (2013). The Genotype-Tissue Expression (GTEx) project. Nat Genet.

[21] Livak KJ, Schmittgen TD (2001). Analysis of relative gene expression data using real-time quantitative PCR and the 2(-Delta Delta C(T)) Method. Methods.

[22] Love MI, Huber W, Anders S (2014). Moderated estimation of fold change and dispersion for RNA-seq data with DESeq2. Genome Biol.

[23] Maechlet M, Rousseeuw P, Struyf A, Hubert M, Hornik K. Cluster: cluster analysis basics and extensions. R package version 2.1.4. 2022. https://cran.r-project.org/web/packages/cluster/citation.html (accessed 20 Dec 2022).

[24] Yu G, Wang L-G, Han Y, He Q-Y (2012). clusterProfiler: an R package for comparing biological themes among gene clusters. OMICS.

[25] Anders S, Reyes A, Huber W (2012). Detecting differential usage of exons from RNA-seq data. Genome Res.

[26] Onecha E, Rapado I, Luz Morales M, Carreño-Tarragona G, Martinez-Sanchez P, Gutierrez X (2021). Monitoring of clonal evolution of acute myeloid leukemia identifies the leukemia subtype, clinical outcome and potential new drug targets for post-remission strategies or relapse. Haematologica.

[27] Onecha E, Linares M, Rapado I, Ruiz-Heredia Y, Martinez-Sanchez P, Cedena T (2019). A novel deep targeted sequencing method for minimal residual disease monitoring in acute myeloid leukemia. Haematologica.

[28] Morales ML, Arenas A, Ortiz-Ruiz A, Leivas A, Rapado I, Rodríguez-García A, et al. MEK inhibition enhances the response to tyrosine kinase inhibitors in acute myeloid leukemia. Sci Rep. 2019;9. 10.1038/s41598-019-54901-9. 10.1038/s41598-019-54901-9 PMC6901485 31819100

[29] Zhang X, Smits AH, van Tilburg GB, Ovaa H, Huber W, Vermeulen M (2018). Proteome-wide identification of ubiquitin interactions using UbIA-MS. Nat Protoc.

[30] Chou TC, Talalay P (1984). Quantitative analysis of dose-effect relationships: the combined effects of multiple drugs or enzyme inhibitors. Adv Enzym Regul.

[31] Cucchi DGJ, Bachas C, van den Heuvel-Eibrink MM, Arentsen-Peters STCJM, Kwidama ZJ, Schuurhuis GJ (2020). Harnessing gene expression profiles for the identification of ex vivo drug response genes in pediatric acute myeloid leukemia. Cancers (Basel).

[32] Ge Y, LaFiura KM, Dombkowski AA, Chen Q, Payton SG, Buck SA (2008). The role of the proto-oncogene ETS2 in acute megakaryocytic leukemia biology and therapy. Leukemia.

[33] Prenkert M, Uggla B, Tidefelt U, Strid H (2010). CRIM1 is expressed at higher levels in drug-resistant than in drug-sensitive myeloid leukemia HL60 cells. Anticancer Res.

[34] Abraham A, Varatharajan S, Karathedath S, Philip C, Lakshmi KM, Jayavelu AK (2015). RNA expression of genes involved in cytarabine metabolism and transport predicts cytarabine response in acute myeloid leukemia. Pharmacogenomics.

[35] Gonçalves V, Henriques AFA, Pereira JFS, Neves Costa A, Moyer MP, Moita LF (2014). Phosphorylation of SRSF1 by SRPK1 regulates alternative splicing of tumor-related Rac1b in colorectal cells. RNA.

[36] Chen S, Lv L, Zhan Z, Wang X, You Z, Luo X (2020). Silencing of long noncoding RNA SRRM2-AS exerts suppressive effects on angiogenesis in nasopharyngeal carcinoma via activating MYLK-mediated cGMP-PKG signaling pathway. J Cell Physiol.

[37] Pellagatti A, Boultwood J (2020). Splicing factor mutant myelodysplastic syndromes: recent advances. Adv Biol Regul.

[38] Dlamini Z, Shoba B, Hull R (2020). Splicing machinery genomics events in acute myeloid leukaemia (AML): in search for therapeutic targets, diagnostic and prognostic biomarkers. Am J Cancer Res.

[39] Zhou X, Li X, Cheng Y, Wu W, Xie Z, Xi Q (2014). BCLAF1 and its splicing regulator SRSF10 regulate the tumorigenic potential of colon cancer cells. Nat Commun.

[40] Whisenant TC, Peralta ER, Aarreberg LD, Gao NJ, Head SR, Ordoukhanian P (2015). The activation-induced assembly of an RNA/protein interactome centered on the splicing factor U2AF2 regulates gene expression in human CD4 T cells. PLoS One.

[41] Miyagawa R, Tano K, Mizuno R, Nakamura Y, Ijiri K, Rakwal R (2012). Identification of cis- and trans-acting factors involved in the localization of MALAT-1 noncoding RNA to nuclear speckles. RNA.

[42] Bradley T, Cook ME, Blanchette M (2015). SR proteins control a complex network of RNA-processing events. RNA.

[43] Hernández F, Pérez M, Lucas JJ, Mata AM, Bhat R, Avila J (2004). Glycogen synthase kinase-3 plays a crucial role in tau exon 10 splicing and intranuclear distribution of SC35. Implications for Alzheimer’s disease. J Biol Chem.

[44] Löwenberg B (2013). Sense and nonsense of high-dose cytarabine for acute myeloid leukemia. Blood.

[45] Di Tullio A, Rouault-Pierre K, Abarrategi A, Mian S, Grey W, Gribben J (2017). The combination of CHK1 inhibitor with G-CSF overrides cytarabine resistance in human acute myeloid leukemia. Nat Commun.

[46] Levin M, Stark M, Berman B, Assaraf TG. Surmounting cytarabine-resistance in acute myeloblastic leukemia cells and specimens with a synergistic combination of hydroxyurea and azidothymidine. Cell Death Dis. 2019;10. 10.1038/s41419-019-1626-x. 10.1038/s41419-019-1626-x PMC6525253 31101804

[47] Jia R, Li C, McCoy JP, Deng C-X, Zheng Z-M (2010). SRp20 is a proto-oncogene critical for cell proliferation and tumor induction and maintenance. Int J Biol Sci.

[48] Karni R, de Stanchina E, Lowe SW, Sinha R, Mu D, Krainer AR (2007). The gene encoding the splicing factor SF2/ASF is a proto-oncogene. Nat Struct Mol Biol.

[49] Cohen-Eliav M, Golan-Gerstl R, Siegfried Z, Andersen CL, Thorsen K, Ørntoft TF (2013). The splicing factor SRSF6 is amplified and is an oncoprotein in lung and colon cancers. J Pathol.

[50] Jensen MA, Wilkinson JE, Krainer AR (2014). Splicing factor SRSF6 promotes hyperplasia of sensitized skin. Nat Struct Mol Biol.

[51] Visconte V, Makishima H, Maciejewski JP, Tiu RV (2012). Emerging roles of the spliceosomal machinery in myelodysplastic syndromes and other hematological disorders. Leukemia.

[52] Hinkle ER, Blue RE, Tsai Y-H, Combs M, Davi J, Coffey AR (2022). Stretching muscle cells induces transcriptional and splicing transitions and changes in SR proteins. Commun Biol.

[53] Muñoz Ú, Puche JE, Hannivoort R, Lang UE, Cohen-Naftaly M, Friedman SL (2012). Hepatocyte growth factor enhances alternative splicing of the Kruppel-like factor 6 (KLF6) tumor suppressor to promote growth through SRSF1. Mol Cancer Res.

[54] Guil S, Cáceres JF (2007). The multifunctional RNA-binding protein hnRNP A1 is required for processing of miR-18a. Nat Struct Mol Biol.

[55] Kozyrev SV, Bernal-Quirós M, Alarcón-Riquelme ME, Castillejo-López C (2012). The dual effect of the lupus-associated polymorphism rs10516487 on BANK1 gene expression and protein localization. Genes Immun.

[56] Yea S, Narla G, Zhao X, Garg R, Tal-Kremer S, Hod E (2008). Ras promotes growth by alternative splicing-mediated inactivation of the KLF6 tumor suppressor in hepatocellular carcinoma. Gastroenterology.

[57] Zhang Z, Lotti F, Dittmar K, Younis I, Wan L, Kasim M (2008). SMN deficiency causes tissue-specific perturbations in the repertoire of snRNAs and widespread defects in splicing. Cell.

[58] Zhou X, Wang R, Li X, Yu L, Hua D, Sun C (2019). Splicing factor SRSF1 promotes gliomagenesis via oncogenic splice-switching of MYO1B. J Clin Invest.

[59] Pimentel H, Parra M, Gee SL, Mohandas N, Pachter L, Conboy JG (2016). A dynamic intron retention program enriched in RNA processing genes regulates gene expression during terminal erythropoiesis. Nucleic Acids Res.

[60] Bohlander SK (2005). ETV6: a versatile player in leukemogenesis. Semin Cancer Biol.

[61] Latorre E, Ostler EL, Faragher RGA, Harries LW (2019). FOXO1 and ETV6 genes may represent novel regulators of splicing factor expression in cellular senescence. FASEB J.

[62] Matera AG, Wang Z (2014). A day in the life of the spliceosome. Nat Rev Mol Cell Biol.

[63] Jeong SSR (2017). Proteins: binders, regulators, and connectors of RNA. Mol Cells.

[64] Casado P, Rodriguez-Prados J-C, Cosulich SC, Guichard S, Vanhaesebroeck B, Joel S (2013). Kinase-substrate enrichment analysis provides insights into the heterogeneity of signaling pathway activation in leukemia cells. Sci Signal.

[65] Schaab C, Oppermann FS, Klammer M, Pfeifer H, Tebbe A, Oellerich T (2014). Global phosphoproteome analysis of human bone marrow reveals predictive phosphorylation markers for the treatment of acute myeloid leukemia with quizartinib. Leukemia.

[66] Murray HC, Enjeti AK, Kahl RGS, Flanagan HM, Sillar J, Skerrett-Byrne DA (2021). Quantitative phosphoproteomics uncovers synergy between DNA-PK and FLT3 inhibitors in acute myeloid leukaemia. Leukemia.

[67] Aasebø E, Berven FS, Bartaula-Brevik S, Stokowy T, Hovland R, Vaudel M, et al. Proteome and phosphoproteome changes associated with prognosis in acute myeloid leukemia. Cancers (Basel). 2020;12. 10.3390/cancers12030709. 10.3390/cancers12030709 PMC7140113 32192169

[68] Nguyen NHK, Wu H, Tan H, Peng J, Rubnitz JE, Cao X, et al. Global proteomic profiling of pediatric AML: a pilot study. Cancers (Basel). 2021;13. 10.3390/cancers13133161. 10.3390/cancers13133161 PMC8268478 34202615

[69] Sciarrillo R, Wojtuszkiewicz A, Assaraf YG, Jansen G, Kaspers GJL, Giovannetti E (2020). The role of alternative splicing in cancer: from oncogenesis to drug resistance. Drug Resist Updat.

[70] Seiler M, Yoshimi A, Darman R, Chan B, Keaney G, Thomas M (2018). H3B-8800, an orally available small-molecule splicing modulator, induces lethality in spliceosome-mutant cancers. Nat Med.

[71] Steensma DP, Wermke M, Klimek VM, Greenberg PL, Font P, Komrokji RS et al. Phase I First-in-Human Dose Escalation Study of the oral SF3B1 modulator H3B-8800 in myeloid neoplasms. Leukemia. 2021. 10.1038/s41375-021-01328-9. 10.1038/s41375-021-01328-9 PMC8632688 34172893

[72] Soncini D, Martinuzzi C, Becherini P, Gelli E, Ruberti S, Todoerti K, et al. Apoptosis reprogramming triggered by splicing inhibitors sensitizes multiple myeloma cells to Venetoclax treatment. Haematologica. 2021. 10.3324/haematol.2021.279276. 10.3324/haematol.2021.279276 PMC9152954 34670358

[73] Ten Hacken E, Valentin R, Regis FFD, Sun J, Yin S, Werner L (2018). Splicing modulation sensitizes chronic lymphocytic leukemia cells to venetoclax by remodeling mitochondrial apoptotic dependencies. JCI Insight.

[74] Moserle L, Ghisi M, Amadori A, Indraccolo S (2010). Side population and cancer stem cells: therapeutic implications. Cancer Lett.

[75] Vazquez R, Breal C, Zalmai L, Friedrich C, Almire C, Contejean A (2021). Venetoclax combination therapy induces deep AML remission with eradication of leukemic stem cells and remodeling of clonal haematopoiesis. Blood Cancer J.

[76] Perez-Riverol Y, Bai J, Bandla C, García-Seisdedos D, Hewapathirana S, Kamatchinathan S (2022). The PRIDE database resources in 2022: a hub for mass spectrometry-based proteomics evidences. Nucleic Acids Res.

