# Supplementary material for: Posttranslational splicing modifications as a key mechanism in cytarabine resistance in acute myeloid leukemia
Source: Leukemia. 2023 Jul 8;37(8):1649–59. doi: 10.1038/s41375-023-01963-4 (PMC10400425; doi:10.1038/s41375-023-01963-4)
Supplement: Supplementary file 1 — Supplemental Material (Leukemia - ProvAccepted) [file 41375_2023_1963_MOESM1_ESM.docx]

**Posttranslational splicing modifications as a key mechanism in cytarabine resistance in Acute Myeloid Leukemia**

*María Luz Morales*, Roberto García-Vicente* et al.*

**SUPPLEMENTARY INFORMATION**

**CONTENT:**

**METHODS**

Cell cultures, patients and healthy donors, and drugs.

Public databases analyses.

RNA and DNA isolation and quantification.

RNA and DNA quality.

RNA expression.

RNA sequencing.

DNA sequencing.

LC-MS/MS analysis.

Immunohistochemistry.

Drug sensitivity assay.

Colony-forming unit assay.

References.

**SUPPLEMENTARY TABLES**

Table S1. Main characteristics of patients and healthy donors whose samples were used in the study.

**SUPPLEMENTARY FIGURES**

Figure S1. *SRSF12* gene expression is altered in AML and other myeloid diseases.

Figure S2. RNA sequencing data analysis.

Figure S3. Levels of phosphorylated SR proteins are elevated during cytarabine resistance.

Figure S4. Levels of phosphorylated SR proteins at diagnosis in responders and non-responders

Figure S5. Genetic alterations in cytarabine resistance

Figure S6. *In vitro* evaluation of cytarabine and splicing inhibitors in cytarabine-sensitive and resistant AML cells.

Figure S7. Generation and validation of cytarabine-resistant OCI-AML3_R cell lines.

Figure S8. *In vitro* evaluation of approved drugs for AML in cytarabine-sensitive and -resistant cells.

Figure S9. Synergistic effect of H3B-8800 and other approved drugs in cytarabine-sensitive and resistant AML cells.

**METHODS**

**Cell cultures, patients and healthy donors, and drugs.**

Human OCI-AML3 and SKM-1 cell lines were obtained from the DSMZ culture collection (Braunschweig, Germany). The human THP-1 cell line was kindly gifted by Vivia Biotech (Tres Cantos, Madrid, Spain). OCI-AML3 cytarabine-resistant cells (OCI-AML3_R) were generated from parental OCI-AML3 after sustained and increasing exposure to cytarabine. All cell lines were cultured in RPMI-1460 medium (ref. BE12-702F/U1, Lonza, Walkersville, MD) supplemented with fetal bovine serum (FBS) (ref. SV30160.03, Cytiva, Marlborough, MA,) and antibiotics (100 U/ml penicillin and 100 µg/ml streptomycin, ref. DE17-602E, Lonza). Culture media for OCI-AML3_R cells were supplemented with a final concentration of 20 µM cytarabine. Cells were grown at 37ºC in a humidified atmosphere containing 5% CO_2_ and maintained by periodic subculture every 2–3 days.

For experiments on primary cells, bone marrow (BM) aspirates from patients with AML or healthy donors were obtained according to standard protocols, and after providing written informed consent in accordance with the Declaration of Helsinki. The main characteristics of the patients and donors whose samples have been used for this study are summarized in Table 1.

Cytarabine (ref. S1648), madrasin (ref. S0280), SPHINX31 (ref. S8810), glasdegib (ref. S7160), midostaurin (ref. S8064), and venetoclax (ref. S8048) were purchased from Selleck Chemicals (Houston, TX). SRPKIN-1 (ref. HY-116856) was bought from MedChemExpress (Monmouth, NJ). H3B-8800 and decitabine were generously provided by Vivia Biotech. Azacitidine was kindly provided by the Pharmacy Department at Hospital 12 de Octubre. Stock solutions of cytarabine, azacitidine and decitabine were dissolved in phosphate buffered saline (PBS) (ref. BE17-516F, Lonza), whereas stock solutions for the other drugs were dissolved in dimethyl sulfoxide (DMSO) (ref. D2650, Sigma-Aldrich, St. Louis, MO), and all were serially diluted in culture medium to obtain the final concentrations for *in vitro* culture. All stock solutions were stored at -80ºC.

All studies involving patient samples were approved by the *Comité Ético de Investigación Clínica* of the *Instituto de Investigación Biomédica* of the *Hospital 12 de Octubre*.

**Public databases analyses**

Using the Gene Expression Profiling Interactive Analysis (GEPIA2) tool ^1^, the mRNA expression levels of genes encoding SR proteins were compared in the tumors of patients from the Acute Myeloid Leukemia project deposited in the public database “The Cancer Genome Atlas (TCGA-LAML, n = 173)” ^2^ and in the bone marrow of healthy controls from the “Genotype-Tissue Expression (GTEx, n = 70) project” ^3^.

**RNA and DNA isolation and quantification**

RNA isolation from bone marrow samples was carried out with TRI Reagent (ref. TR118, Molecular Research Center, Cincinnati, OH) following standard clinical protocols, whereas RNA extraction from healthy donors and DNA isolation from AML cell lines were performed using the AllPrep DNA/RNA/Protein Mini Kit (Qiagen, Hilden, Germany). DNA extraction from patients with AML was performed in a Maxwell®16 MDx instrument (Promega Biotech Iberica, S.L.).

RNA was quantified on a Nanodrop Spectrophotometer ND-1000 (ThermoFisher Scientific, Waltham, MA). DNA quantification (technical duplicates) was performed with the Qubit 4.0 fluorometer and corresponding reagents (Qubit dsDNA BR assay kit [ref. Q32853] and Qubit dsDNA HS assay kit [ref. Q32854], both from Invitrogen ThermoFisher).

**RNA and DNA quality**

RNA quality of samples used for RNA sequencing was determined using the Bioanalyzer 2100 (ref. G2939B, Agilent Technologies, Las Rozas, Spain) and the Agilent RNA 6000 pico kit (ref. 5067-1513, Agilent Technologies). According to the integrity of RNA samples some modifications were applied during RNA library generation (with a threshold integrity of 200 bp). The quality of DNA libraries was evaluated in a Bioanalyzer 2100 using Agilent DNA 1000 reagents kit (ref. 5067-1504, Agilent Technologies) or High Sensitivity DNA reagents kit (ref. 5067-4626, Agilent Technologies).

**RNA expression**

Reverse transcription polymerase chain reaction (RT-PCR)

cDNA synthesis from mRNA was performed using the High Capacity cDNA Reverse Transcription Kit (ref. 4374967, Applied Biosystems, ThermoFisher Scientific) on the Veriti™ 96-Well Thermal Cycler (ref. 4375786, ThermoFisher Scientific). 500 ng of RNA was used, maintaining a 1:1 ratio of reverse transcriptase reaction mix and RNA, and applying the following program: 10 minutes at 25°C, 120 minutes at 37°C and 5 minutes at 85°C.

Real-time polymerase chain reaction (qPCR)

Gene expression levels of *SRRM2* (Unique Assay ID: qHsaCED0046512, Bio-Rad Laboratories, Hercules), *SRSF12* (Unique Assay ID: qHsaCED0045641, Bio-Rad Laboratories) and β-glucuronidase (ref. 4304970, ThermoFisher Scientific) gene (*GUS*) as a control, were measured using the TaqMan Gene Expression Master Mix (ref. 4369016, ThermoFisher Scientific) and the probe SYBR™ Green I Nucleic Acid Gel Stain (ref. S7563, ThermoFisher Scientific). Samples from AML (n = 16, patients 1–16 for *SRRM2* and *SRSF12* quantification; and n = 7, patients 10–15 and 17 for quantification of *SRRM2* in paired samples), MPN (n = 12, patients N1–N12), MDS (n = 11, patients S1–S11) patients and healthy controls (n = 15, controls 1–15) (Table 1) were measured in triplicate in the QuantStudio 5 System (ref. A28568, Applied Biosystems, ThermoFisher Scientific) using the following conditions: 2 minutes at 50ºC, 10 minutes at 95ºC and 40× (15 seconds at 95ºC and 1 minute at 60ºC). Gene expression was quantified using the comparative ΔΔCt method ^4^.

**RNA sequencing**

RNA libraries of paired samples (diagnosis and cytarabine-resistance) of 25 patients with AML (Table 1) were generated using the KAPA RNA HyperPrep kit with RiboErase protocol (HMR) (KR1351-v1.16) for the Illumina platform and the reagents from the kits KAPA RNA HyperPrep (ref. 08105952001, Roche), KAPA HyperPlus (ref. 07962380001, Roche), KAPA Pure Beads (ref. 07983280001, Roche), and SeqCap Adapter B (ref. 07141549001, Roche) and the ProFlex™ Base system (ref. 4483636, Applied Biosystems). RNA libraries were quantified using the KAPA Library Quant kit (Illumina) ROX Low qPCR Mix (ref. 07960336001, Roche) and the QuantStudio™ 5 Real-Time PCR Instrument (96-well 0.1 ml block) (ref. A28133, Applied Biosystems). Finally, libraries were sequenced on the NextSeq 500/550 System (Illumina).

Paired-end raw reads from RNAseq runs were aligned to the reference human genome GRCh38.p13 using STAR v2.7.9a ^5^, and gene-level quantifications for GENCODE V38 annotation. Downstream and statistical analyses were carried out using R v4.0.3 (R Core Team, 2020). Differential gene expression (DGE) analysis was performed using DESeq2 v1.30.1 ^6^ and implementing the design formula ~ Patient_ID + Moment_of_sample + blasts. Blast cytology count per sample was introduced as a factor to control for the effect of blasts which was detected as a source of variability in the data (Figure S3D). Low-expressed genes, with a median value across all samples equal or lower than 10 counts and sexual chromosome genes were discarded from the raw gene count matrix. We performed a paired analysis by contrasting post-treatment to diagnosis –two time-points per patient– separately for each response groups either relapse (RL) or refractory (RE). For the subsequent analyses, blast count effect was corrected using the removeBatchEffect() function from the limma v3.46.0 R package ^7^. We performed a gene clustering analysis based on the partitioning around medoids (PAM) algorithm using cluster v2.1.0 R package ^8^, across 4 different sample groups (RL_Dx, RL_Tr, RE_Dx, RE_Tr). For the transcription factor (TF) analyses, TF enrichment was determined by calculating the overlap of known genomic regions of the TF binding sites with the genomic regions of gene promoters from the cluster 4, using the ReMapEnrich v0.99.0 R package ^9^. We also estimated TF regulatory activities by applying the WMEAN method to gene expression values from TF regulons (confidence levels A-C) described in the decoupleR v2.3.2 R package data ^10^. Finally, gene ontology (GO) overrepresentation analyses were performed using the clusterProfiler v3.18.1 R package ^11^, defining biological processes as the GO terms of interest. Differential exon usage analysis was carried out by Dreamgenics S.L. (Oviedo, Spain) using the DEXseq package ^12^.

**DNA sequencing**

Variants in the DNA sequence of parental (OCI-AML3) and cytarabine-resistant (OCI-AML3_R) cell lines (three independent replicates), and from patients with AML at diagnosis (see “mutations” in Table 1) were studied with a customized NGS myeloid panel of 32 genes frequently mutated in myeloid diseases ^13,14^. DNA library generation was carried out with the Ion Ampliseq™ Library Kit 2.0 - 96 LV (ref. 4480441, Ion Torrent, ThermoFisher Scientific), using Ion Xpress™ barcode adapters (ref. 4471250, 4474009, 4474518, 4474519, 4474520 and 4474521 from Ion Torrent), and Ampure XP (ref. A63881, Beckman Coulter, Life Sciences, Spain) on the 2720 Thermal cycler (ref. 4359659, Applied Biosystems). DNA libraries were quantified with the QIAseq library quant assay kit (ref. 333314, Qiagen) for the QuanStudio™ 5 Real-Time PCR Instrument (96-well 0.1 ml block) (ref. A28133, Applied Biosystems). Finally, 2 million reads for each sample were collected in the ION OneTouch2 System (ref. 4474779, Thermo Fisher Scientific). Ion Reporter and Integrative Genome Viewer (v. 2.8.12, Broad Institute, University of California) were used for further analysis.

**LC-MS/MS analysis**

Phosphoproteomic analysis was conducted at the Proteomics Core Unit of the *Centro Nacional de Investigaciones Oncológicas* (CNIO). Cells were lysed using 7 M urea, 2 M thiourea, in 100 mM Hepes pH 7.5, supplemented with 1:1000 (v/v) of benzonase (Novagen) and 1:100 (v/v) of Halt^TM^ phosphatase and protease inhibitor cocktail 100x (Thermo Fisher Scientific). Then, samples were digested by means of the standard FASP protocol. Briefly, proteins were reduced and alkylated (15 mM TCEP, 50 mM CAA, 30 min in the dark, RT) and sequentially digested with Lys-C (Wako) (protein:enzyme ratio 1:100, o/n at RT) and trypsin (Promega) (protein:enzyme ratio 1:100, 6 h at 37 °C). Resulting peptides were desalted using a Sep-Pak C18 cartridge. Alternatively, samples were solubilized 10 minutes at 95 °C in 5% SDS, 50 mM TEAB pH 7.55. Then, samples were digested by means of the Protifi™ S-Trap™ Mini Spin Column Digestion Protocol. Briefly, proteins were reduced and alkylated (15 mM TCEP, 25 mM CAA) 1h at 45 °C in the dark. SDS was removed from samples in the S-Trap column using 90% methanol in 100 mM TEAB and proteins were digested with 125 µl of trypsin in 50 mM TEAB pH 7.55 (trypsin (Promega), protein:enzyme ratio 1:100, 1 h at 47 °C). Resulting peptides were desalted using a Sep-Pak C18 cartridge.

Phosphopeptides were enriched using home-made TiO_2_ micro-columns. Briefly, peptides were resuspended in 6% TFA and 80% CH3CN and incubated for 20 min with TiO_2_ beads (10 µm particle size) (GL-Science) using a sample:TiO_2_ ratio of 1:2. Unbound peptides were kept for further analysis. Prior to incubation, TiO_2_ beads were pre-conditioned with a solution of 20 mg/mL DHB in 80% CH_3_CN 6% TFA for 20 min. Then, beads were sequentially washed with 100 µL of 6% TFA and 10% CH_3_CN, 100 µL of 6% TFA and 100 µL of 40% CH_3_CN and 6% TFA and 60% CH_3_CN. Finally, phosphopeptides were eluted first with 20 µL of 5% NH_4_OH and then with 20 µL 5% NH4OH in 10% CH3CN in the same vial. Samples were speed-vac dried, re-dissolved 0.5% formic acid and analyzed without further desalting. Unbound peptides were desalted using C18 stage-tips, speed-vac dried and re-dissolved in 0.5% formic acid. Each sample was analyzed twice by LC-MS/MS.

LC-MS/MS was done by coupling a nanoLC-Ultra 1D+ system (Eksigent) to an Impact mass spectrometer (Bruker) via a Captivespray source (Bruker) supplemented with a nanoBooster operated at 0.2 bar/min with isopropanol as dopant. Peptides were loaded into a trap column (NS-MP-10 BioSphere C18 5 µm, 20 mm length, Nanoseparations) for 10 min at a flow rate of 2.5 µl/min in 0.1% FA. Then peptides were transferred to an analytical column (ReproSil Pur C18-AQ 1.9 µm, 400 mm length and 0.075 mm ID) and separated using a curved gradient (buffer A: 4% ACN, 0.1% FA; buffer B: 100% ACN, 0.1% FA) at a flow rate of 250 nL/min from 2% to 35.2% of buffer B. The effective gradient time was 90 min for enriched phosphopeptide samples and 120min for the unbound fraction samples.The peptides were electrosprayed (1.35 kV) into the mass spectrometer with a heated capillary temperature of 180 °C. The mass spectrometer was operated in a data-dependent mode, with an automatic switch between MS (80-1600 m/z) and MS/MS (80-1600 m/z) scans using a top 20 method (threshold signal ≥ 500 counts, z ≥ 2 and m/z ≥ 350). An active exclusion of 60 sec was used. The precursor intensities were re-evaluated in the MS scan (n) regarding their values in the previous MS scan (n-1). Any m/z intensity exceeding 5 times the measured value in the preceding MS scan was reconsidered for MS/MS. Peptides were isolated using a 2 Th window and fragmented using collision induced dissociation (CID) with a collision energy of 23-56 eV as function of the m/z value.

Raw files were processed with MaxQuant using the standard settings against a human protein database (UniProtKB/Swiss-Prot, 20,187 sequences) supplemented with contaminants. Carbamidomethylation of cysteines was set as a fixed modification whereas oxidation of methionines, protein N-term acetylation and phosphorylation of serines, threnonines and tyrosines were set as variable modifications. Minimal peptide length was set to 7 amino acids and a maximum of two tryptic missed-cleavages were allowed. Results were filtered at 1% FDR (peptide and protein level).

Differential phophoproteomics analysis was performed using the DEP v1.12.0 R package ^15^, to establish unique peptide identifiers with the make_unique() function, and limma v3.46.0 R package ^7^, using the duplicateCorrelation() function to account for within-patient correlations, fitting a linear model to the phosphoproteomic data with the lmFit() function, and applying empirical Bayes moderated t statistics with eBayes() function to estimate differences in phosphopeptide abundance. Comparisons between conditions were assessed by applying the design formula ~ Condition.

**Immunohistochemistry**

Methanol-fixed bone marrow smears from patients with AML at diagnosis (n = 64), resistance (n = 4) or after cytarabine treatment (n = 3) (Table 1) were obtained according to standard protocols. Smears were refreshed in PBS/1% bovine serum albumin (BSA, ref. A2153, Sigma-Aldrich), and endogenous peroxidase was inactivated by 5 minutes incubation with 3% hydrogen peroxide. Antigen unmasking and recovery was performed by heat-mediated antigen retrieval with a citrate buffer (citric acid 1.8 mM, sodium citrate 8.2 mM). Next, samples were blocked with PBS/5%BSA for one hour to prevent non-specific binding and were incubated overnight with the anti-SR protein family (MABE126, Sigma-Aldrich) or anti-phosphoepitope SR proteins (MABE50, Sigma-Aldrich) antibodies. Signals were detected with an anti-mouse horseradish peroxidase-conjugated secondary antibody (ref. #8125, Cell Signaling Technology, Danvers, MA) and the DAB substrate kit (ab94665, Abcam, Cambridge, UK). Slides were counterstained with Carazzi's hematoxylin solution (ref. 255298.1610, PanReac AppliChem, Ottoweg, Darmstadt, Germany) before mounting in DPX Mountant for histology (ref. 06522, Sigma-Aldrich). Slide images were taken with a digital camera (Leica ICC50 W) connected to a Leica DM 2000 LED microscope (Leica Microsystems, Wetzlar, Germany). Blast staining was quantified from 40× fields randomly chosen, until reaching a cellularity of 100–400 blasts, and averaged. In bone marrow smears, each blast was classified according to its staining, negative or positive, and location, nuclear or cytoplasmic. ImageJ 1.53 (University of Wisconsin) was used to analyze the ratio of normalized staining.

**Drug sensitivity assay**

*In vitro* growth analyses after monotherapy or combination treatments were performed by seeding 3×10^4^ OCI-AML3, SKM-1, THP-1 or OCI-AML3_R cells per well into 96-microwell plates. For *ex vivo* experiments, bone marrow mononuclear cells (BMMC) were obtained by standard density gradient centrifugation on Ficoll cushions (ref. 17144002, Cytiva), and 2×10^4^ mononuclear cells were seeded per well in StemSpan SFEM II medium (ref. 09605, StemCell Technologies, Grenoble, France) supplemented with 1× StemSpan™ CC100 (ref. 02690, StemCell Technologies). Cells were exposed to different drug doses (Table 2) and cell viability was determined after 48 or 72 hours (*in vitro* or *ex vivo*) of exposure to drugs or vehicle using the Cell Counting Kit-8 reagent from Sigma-Aldrich (ref. 96992) and measured with the Epoch microplate reader running Gen5 software. Cell survival percentages from 3 independent experiments were calculated and normalized to controls. Half maximal inhibitory concentration (IC_50_) values were determined according to nonlinear regression using GraphPad Prism 5.01 (La Jolla, CA) and dose-response graphics were represented as the mean survival ± standard error of the mean (SEM). The combination index (CI) and normalized isobolograms were obtained using Compusyn software (Combosyn Inc., Paramus, NJ) ^16^.

**Colony-forming unit assay**

To test for treatment-related toxicity, colony-forming unit assays of CD34+ progenitor cells from healthy donors (n = 3, C16–C18, Table 1) were performed. CD34+ cells were isolated from BMMCs with the MACs CD34 MicroBead Kit (ref. 130-046-703, Miltenyi Biotec S.L.). 2×10^3^ cells were resuspended in 200 μl of IMDM medium supplemented with 25 mM HEPES (ref. BE12-722F, Lonza) and 20% FBS and were then submerged in 3.8 ml of methylcellulose (Methocult Express; ref. 4437, StemCell Technologies) with different concentrations of the corresponding drugs (H3B-8800, venetoclax or their combination) or DMSO as control. Doses ranging from 0.4 µM to 2 µM of venetoclax were combined with doses of 0.01 µM and 0.001 µM of H3B-8800. Cells were plated in 35-mm Petri dishes in triplicates of 1 ml and incubated at 37ºC in a humidified atmosphere containing 5% CO_2_. Colony-forming units (CFU-granulocyte-monocyte and erythroid colonies) were scored at day 14 using a stereomicroscope Olympus VMZ 1 x – 4x (Olympus Iberia S.A.U., Barcelona, Spain) and captured using ChemiDoc Imaging Systems (Bio-Rad Laboratories).

**References**

1 Tang Z, Li C, Kang B, Gao G, Li C, Zhang Z. GEPIA: a web server for cancer and normal gene expression profiling and interactive analyses. *Nucleic Acids Res* 2017; **45**: W98–W102.

2 Ley TJ, Miller C, Ding L, Raphael BJ, Mungall AJ, Robertson AG *et al.* Genomic and epigenomic landscapes of adult de novo acute myeloid leukemia. *N Engl J Med* 2013; **368**: 2059–2074.

3 GTEx Consortium. The Genotype-Tissue Expression (GTEx) project. *Nat Genet* 2013; **45**: 580–585.

4 Livak KJ, Schmittgen TD. Analysis of relative gene expression data using real-time quantitative PCR and the 2(-Delta Delta C(T)) Method. *Methods* 2001; **25**: 402–408.

5 Dobin A, Davis CA, Schlesinger F, Drenkow J, Zaleski C, Jha S *et al.* STAR: ultrafast universal RNA-seq aligner. *Bioinformatics* 2013; **29**: 15–21.

6 Love MI, Huber W, Anders S. Moderated estimation of fold change and dispersion for RNA-seq data with DESeq2. *Genome Biology* 2014; **15**: 550.

7 Ritchie ME, Phipson B, Wu D, Hu Y, Law CW, Shi W *et al.* limma powers differential expression analyses for RNA-sequencing and microarray studies. *Nucleic Acids Research* 2015; **43**: e47.

8 Maechler M, Rousseeuw P, Struyf A, Hubert M, Hornik K. cluster: Cluster Analysis Basics and Extensions. 2022. https://CRAN.R-project.org/package=cluster.

9 Hammal F, de Langen P, Bergon A, Lopez F, Ballester B. ReMap 2022: a database of Human, Mouse, Drosophila and Arabidopsis regulatory regions from an integrative analysis of DNA-binding sequencing experiments. *Nucleic Acids Research* 2022; **50**: D316–D325.

10 Badia-i-Mompel P, Vélez J, Braunger J, Geiss C, Dimitrov D, Müller-Dott S *et al.* decoupleR: Ensemble of computational methods to infer biological activities from omics data. 2021; : 2021.11.04.467271.

11 Yu G, Wang L-G, Han Y, He Q-Y. clusterProfiler: an R package for comparing biological themes among gene clusters. *OMICS* 2012; **16**: 284–287.

12 Anders S, Reyes A, Huber W. Detecting differential usage of exons from RNA-seq data. *Genome Res* 2012; **22**: 2008–2017.

13 Onecha E, Rapado I, Luz Morales M, Carreño-Tarragona G, Martinez-Sanchez P, Gutierrez X *et al.* Monitoring of clonal evolution of acute myeloid leukemia identifies the leukemia subtype, clinical outcome and potential new drug targets for post-remission strategies or relapse. *Haematologica* 2021; **106**: 2325–2333.

14 Onecha E, Linares M, Rapado I, Ruiz-Heredia Y, Martinez-Sanchez P, Cedena T *et al.* A novel deep targeted sequencing method for minimal residual disease monitoring in acute myeloid leukemia. *Haematologica* 2019; **104**: 288–296.

15 Zhang X, Smits AH, van Tilburg GB, Ovaa H, Huber W, Vermeulen M. Proteome-wide identification of ubiquitin interactions using UbIA-MS. *Nat Protoc* 2018; **13**: 530–550.

16 Chou TC, Talalay P. Quantitative analysis of dose-effect relationships: the combined effects of multiple drugs or enzyme inhibitors. *Adv Enzyme Regul* 1984; **22**: 27–55.

**SUPPLEMENTAL TABLES**

**Table S1. Main characteristics of patients and healthy donors whose samples were used in the study.**

|  |  |  |  |  |  |  |  |  | **TECHNIQUE** | | | | | | | | |
| --- | --- | --- | --- | --- | --- | --- | --- | --- | --- | --- | --- | --- | --- | --- | --- | --- | --- |
| **ID** | **G** | **A** | **F** | **MUTATIONS** | **C-R** | **M** | **TYPE** | **%** | **1** | **2** | **3** | **4** | **5** | **6** | **7** | **8** | **9** |
| **1** | F | 30 | 0 | RUNX1, TET2 |  | DG | RNA | 90 | X |  |  |  |  |  |  |  |  |
| **2** | F | 68 | 5 | NS |  | DG | RNA | 86 | X |  | X |  |  |  |  |  |  |
| **3** | M | 63 | 1 | FLT3-L576Q |  | DG | RNA | 93 | X |  | X |  |  |  |  |  |  |
| **4** | M | 42 | 1 | FLT3-ITD |  | DG | RNA | 91 | X |  | X |  |  |  |  |  |  |
| **5** | M | 79 | 4 | NPM1 |  | DG | RNA | 51 | X |  | X |  |  |  |  |  |  |
| **6** | M | 72 | 6 |  | NR | DG | RNA  MET | 18 | X |  | X |  |  |  | X |  |  |
| **7** | M | 62 | 4 | KMT2A, NRAS, TP53 | NR | DG | RNA  MET | 29 | X |  | X |  |  | X | X |  |  |
|  |  |  |  |  |  | ACT | MET | 38 |  |  |  |  |  | X |  |  |  |
| **8** | F | 84 | 1 | NPM1 | R | DG | RNA  MET | 92 | X |  | X |  |  |  | X |  |  |
| **9** | F | 38 | 1 | IDH1, KIT |  | DG | RNA | 65 | X |  | X |  |  |  |  |  |  |
| **10** | F | 80 | 1 | CEBPA | NR | DG | RNA  MET  BMMC | 95 | X |  | X | X |  |  | X |  |  |
|  |  |  |  |  |  | RES | BMMC | 90 |  |  |  | X |  |  |  |  |  |
| **11** | F | 65 | 5 | TET2 | NR | DG | RNA  MET | 50 | X |  | X |  |  |  | X |  |  |
| **12** | F | 36 | 2 | FLT3-ITD, NPM1 | R | DG | RNA  MET | 77 | X |  | X |  |  |  | X |  |  |
| **13** | M | 43 | 4 |  | NR | DG | RNA  MET | 26 | X |  | X |  |  |  | X |  |  |
| **14** | F | 76 | 1 | ASXL1, IDH1, KIT, RUNX1, TET2 | R | DG | RNA  MET | 88 | X |  | X |  |  |  | X |  |  |
| **15** | F | 56 | 1 | DNMT3A, EZH2, IDH2, TET2 | R | DG | RNA  MET | 88 | X |  | X |  |  |  | X |  |  |
| **16** | F | 74 | 1 | DNMT3A | R | DG | RNA  MET | 66 | X |  | X |  | X |  | X |  |  |
|  |  |  |  |  |  | RES | MET | 53 |  |  |  |  | X |  |  |  |  |
| **17** | M | 66 | 1 | CEBPA, SF3A1, U2AF1 | R | DG | RNA  MET | 90 |  |  | X |  | X |  | X |  |  |
|  |  |  |  |  |  | RES | MET | 94 |  |  |  |  | X |  |  |  |  |
| **18** | M | 24 | 5 | KDM6A, KMT2A | NR | DG | MET  BMMC | 86 |  |  |  | X |  |  | X |  |  |
|  |  |  |  |  |  | RES | BMMC | 77 |  |  |  | X |  |  |  |  |  |
| **19** | F | 47 | 2 | DNMT3A, EZH2, FLT3, IDH1, KDM6A, KIT, KMT2A, RUNX1, TET2 | R | DG | MET  BMMC | 64 |  |  |  | X |  |  | X |  |  |
|  |  |  |  |  |  | RES | BMMC | 63 |  |  |  | X |  |  |  |  |  |
| **20** | F | 64 | 1 | DNMT3A, JAK2, KDM6A, KIT, RUNX1, SF3B1, VHL | R | DG | MET | 70 |  |  |  |  | X |  | X |  |  |
|  |  |  |  |  |  | RES | MET | 18 |  |  |  |  | X |  |  |  |  |
| **21** | M | 71 | 1 | DNMT3A, EZH2 | R | DG | MET | 54 |  |  |  |  | X |  | X |  |  |
|  |  |  |  |  |  | RES | MET | 35 |  |  |  |  | X |  |  |  |  |
| **22** | M | 60 | 1 | IDH2, NPM1 | NR | DG | MET | 65 |  |  |  |  |  | X | X |  |  |
|  |  |  |  |  |  | ACT | MET | 58 |  |  |  |  |  | X |  |  |  |
| **23** | F | 53 | 1 | IDH2, PHF6, STAG2 | NR | DG | MET | 89 |  |  |  |  |  | X | X |  |  |
|  |  |  |  |  |  | ACT | MET | 83 |  |  |  |  |  | X |  |  |  |
| **24** | F | 55 | 0 |  | R | DG | MET | 80 |  |  |  |  |  |  | X |  |  |
| **25** | M | 64 | 5 | IDH1, NRAS, VHL | R | DG | MET | 72 |  |  |  |  |  |  | X |  |  |
| **26** | M | 47 | 1 | JAK2, KMT2A | R | DG | MET | 32 |  |  |  |  |  |  | X |  |  |
| **27** | F | 35 | 1 | TP53 | R | DG | MET | 87 |  |  |  |  |  |  | X |  |  |
| **28** | F | 36 | 4 | CBL, FLT3, IDH1, KIT, NRAS, ZRSR2 | R | DG | MET | 78 |  |  |  |  |  |  | X |  |  |
| **29** | F | 37 | 2 | DNMT3A, FLT3-ITD, TP53 | R | DG | MET | 73 |  |  |  |  |  |  | X |  |  |
| **30** | F | 60 | 1 | IDH1, FLT3-ITD, JAK2 | R | DG | MET | 55 |  |  |  |  |  |  | X |  |  |
| **31** | F | 46 | 4 | DNMT3A, JAK2, KRAS | R | DG | MET | 53 |  |  |  |  |  |  | X |  |  |
| **32** | F | 81 | s | NS | R | DG | MET | 38 |  |  |  |  |  |  | X |  |  |
| **33** | F | 71 | 2 | FLT3-ITD, NPM1 | R | DG | RNA  MET | 70 |  |  | X |  |  |  | X |  |  |
| **34** | F | 54 | 1 | IDH2, TET2 | R | DG | MET | 67 |  |  |  |  |  |  | X |  |  |
| **35** | M | 60 | 4 |  | R | DG | MET | 54 |  |  |  |  |  |  | X |  |  |
| **36** | M | 34 | 2 | ASXL1, DNMT3A, IDH1 | R | DG | MET | 42 |  |  |  |  |  |  | X |  |  |
| **37** | F | 68 | 1 | IDH2, NRAS | R | DG | MET | 63 |  |  |  |  |  |  | X |  |  |
| **38** | F | 59 | 5 | CBL, FLT3, KRAS, NPM1 | R | DG | BMMC  MET | 71 |  |  |  |  |  |  | X | X |  |
| **39** | F | 57 | 5 | KIT, NRAS | R | DG | MET | 78 |  |  |  |  |  |  | X |  |  |
| **40** | M | 49 | 2 |  | R | DG | MET | 52 |  |  |  |  |  |  | X |  |  |
| **41** | F | 75 | s | KMT2A, RUNX1 | R | DG | MET | 31 |  |  |  |  |  |  | X |  |  |
| **42** | F | 67 | s | TET2 | R | DG | MET | 70 |  |  |  |  |  |  | X |  |  |
| **43** | M | 50 | 5 | DNMT3A, NPM1 | R | DG | BMMC  MET | 78 |  |  |  |  |  |  | X | X |  |
| **44** | M | 49 | 6 | MPL, SETBP1 | R | DG | MET | 83 |  |  |  |  |  |  | X |  |  |
| **45** | M | 73 | 5 |  | R | DG | MET | 63 |  |  |  |  |  |  | X |  |  |
| **46** | M | 63 | 5 | FLT3, IDH2, NPM1 | R | DG | MET | 59 |  |  |  |  |  |  | X |  |  |
| **47** | F | 63 | 1 | KIT, RUNX1 | R | DG | MET | 30 |  |  |  |  |  |  | X |  |  |
| **48** | F | 64 | t | JAK2, KIT, NPM1, NRAS | R | DG | MET | 30 |  |  |  |  |  |  | X |  |  |
| **49** | F | 62 | 5 | KMT2A | R | DG | MET | 93 |  |  |  |  |  |  | X |  |  |
| **50** | M | 74 | 2 | CEBPA, RUNX1, SRSF2, STAG2, TET2 | R | DG | MET | 34 |  |  |  |  |  |  | X |  |  |
| **51** | M | 50 | 4 | FLT3, IDH2, NRAS, WT1 | R | DG | MET | 65 |  |  |  |  |  |  | X |  |  |
| **52** | F | 48 | t | EPAS1, FLT3, NRAS, RUNX1 | R | DG | MET | 28 |  |  |  |  |  |  | X |  |  |
| **53** | F | 55 | 2 | FLT3-ITD | R | DG | MET | 71 |  |  |  |  |  |  | X |  |  |
| **54** | M | 44 | 1 | ETV6, RUNX1 | R | DG | MET | 86 |  |  |  |  |  |  | X |  |  |
| **55** | M | 66 | 4 | IDH2, NPM1 | R | DG | MET | 28 |  |  |  |  |  |  | X |  |  |
| **56** | F | 71 | 5 | DNMT3A, FLT3, NPM1, SMC1A | R | DG | MET | 84 |  |  |  |  |  |  | X |  |  |
| **57** | F | 44 | 5 | CBL, NRAS | R | DG | MET | 80 |  |  |  |  |  |  | X |  |  |
| **58** | M | 76 | 2 | NS | NR | DG | MET | 41 |  |  |  |  |  |  | X |  |  |
| **59** | M | 76 | 2 | IDH1, JAK2, KIT, TP53 | NR | DG | MET | 59 |  |  |  |  |  |  | X |  |  |
| **60** | M | 67 | s |  | NR | DG | MET | 27 |  |  |  |  |  |  | X |  |  |
| **61** | M | 77 | 1 | CBL, FLT3-ITD, SF3A1 | NR | DG | MET | 86 |  |  |  |  |  |  | X |  |  |
| **62** | M | 47 | t | TP53 | NR | DG | RNA  MET | 26 |  |  | X |  |  |  | X |  |  |
| **63** | M | 51 | 2 | RUNX1, TP53 | NR | DG | RNA  MET | 21 |  |  | X |  |  |  | X |  |  |
| **64** | M | 81 | t | NRAS | NR | DG | MET | 21 |  |  |  |  |  |  | X |  |  |
| **65** | M | 65 | s |  | NR | DG | RNA  MET | 81 |  |  | X |  |  |  | X |  |  |
| **66** | M | 59 | s | RAD21, SF3B1, TET2 | NR | DG | MET | 20 |  |  |  |  |  |  | X |  |  |
| **67** | F | 56 | 2 | BCORL1, DNMT3A, FLT3, NRAS, RUNX1 | NR | DG | MET | 53 |  |  |  |  |  |  | X |  |  |
| **68** | M | 58 | 1 | DNMT3A, IDH2, NPM1, RAD21 | NR | DG | BMMC  MET | 79 |  |  |  |  |  |  | X | X |  |
| **69** | M | 59 | 1 | BCORL1, CALR, NPM1, TET2 | NR | DG | MET | 88 |  |  |  |  |  |  | X |  |  |
| **70** | M | 56 | 2 | TP53 | NR | DG | MET | 40 |  |  |  |  |  |  | X |  |  |
| **71** | M | 73 | 5 | NS |  | DG | RNA | 91 |  |  | X |  |  |  |  |  |  |
| **72** | F | 63 | 5 | ASXL1, SF3B1 |  | DG | RNA | 55 |  |  | X |  |  |  |  |  |  |
| **73** | M | 4 | 5 | NS |  | DG | RNA | 34 |  |  | X |  |  |  |  |  |  |
| **74** | M | 48 | 2 | DNMT3A, IDH2, KRAS, NRAS, TET2 |  | DG | RNA | 56 |  |  | X |  |  |  |  |  |  |
| **75** | F | 1 | 5 | FLT3-ITD, NPM1, NRAS |  | DG | RNA | 74 |  |  | X |  |  |  |  |  |  |
| **N1** | M | 75 |  |  |  |  | RNA |  | X | X |  |  |  |  |  |  |  |
| **N2** | M | 53 |  |  |  |  | RNA |  | X | X |  |  |  |  |  |  |  |
| **N3** | M | 58 |  |  |  |  | RNA |  | X | X |  |  |  |  |  |  |  |
| **N4** | M | 79 |  |  |  |  | RNA |  | X | X |  |  |  |  |  |  |  |
| **N5** | F | 83 |  |  |  |  | RNA |  | X | X |  |  |  |  |  |  |  |
| **N6** | F | 74 |  |  |  |  | RNA |  | X | X |  |  |  |  |  |  |  |
| **N7** | F | 44 |  |  |  |  | RNA |  | X | X |  |  |  |  |  |  |  |
| **N8** | M | 88 |  |  |  |  | RNA |  | X | X |  |  |  |  |  |  |  |
| **N9** | M | 75 |  |  |  |  | RNA |  | X | X |  |  |  |  |  |  |  |
| **N10** | F | 81 |  |  |  |  | RNA |  | X | X |  |  |  |  |  |  |  |
| **N11** | M | 87 |  |  |  |  | RNA |  | X | X |  |  |  |  |  |  |  |
| **N12** | M | 73 |  |  |  |  | RNA |  | X |  |  |  |  |  |  |  |  |
| **S1** | M | 64 |  |  |  |  | RNA |  | X | X |  |  |  |  |  |  |  |
| **S2** | M | 82 |  |  |  |  | RNA |  | X | X |  |  |  |  |  |  |  |
| **S3** | M | 59 |  |  |  |  | RNA |  | X | X |  |  |  |  |  |  |  |
| **S4** | M | 66 |  |  |  |  | RNA |  | X | X |  |  |  |  |  |  |  |
| **S5** | M | 73 |  |  |  |  | RNA |  | X | X |  |  |  |  |  |  |  |
| **S6** | M | 78 |  |  |  |  | RNA |  | X | X |  |  |  |  |  |  |  |
| **S7** | F | 42 |  |  |  |  | RNA |  | X | X |  |  |  |  |  |  |  |
| **S8** | F | 79 |  |  |  |  | RNA |  | X | X |  |  |  |  |  |  |  |
| **S9** | M | 80 |  |  |  |  | RNA |  | X | X |  |  |  |  |  |  |  |
| **S10** | F | 84 |  |  |  |  | RNA |  | X | X |  |  |  |  |  |  |  |
| **S11** | M | 88 |  |  |  |  | RNA |  | X | X |  |  |  |  |  |  |  |
| **C1** | F | 68 |  |  |  |  | RNA |  | X | X |  |  |  |  |  |  |  |
| **C2** | M | 59 |  |  |  |  | RNA |  | X | X |  |  |  |  |  |  |  |
| **C3** | M | 68 |  |  |  |  | RNA |  | X | X |  |  |  |  |  |  |  |
| **C4** | F | 59 |  |  |  |  | RNA |  | X | X |  |  |  |  |  |  |  |
| **C5** | M | 82 |  |  |  |  | RNA |  | X | X |  |  |  |  |  |  |  |
| **C6** | M | 33 |  |  |  |  | RNA |  | X | X |  |  |  |  |  |  |  |
| **C7** | F | 54 |  |  |  |  | RNA |  | X | X |  |  |  |  |  |  |  |
| **C8** | F | 55 |  |  |  |  | RNA |  | X | X |  |  |  |  |  |  |  |
| **C9** | M | 36 |  |  |  |  | RNA |  | X | X |  |  |  |  |  |  |  |
| **C10** | F | 80 |  |  |  |  | RNA |  | X | X |  |  |  |  |  |  |  |
| **C11** | M | 52 |  |  |  |  | RNA |  | X | X |  |  |  |  |  |  |  |
| **C12** | F | 72 |  |  |  |  | RNA |  | X | X |  |  |  |  |  |  |  |
| **C13** | F | 28 |  |  |  |  | RNA |  | X | X |  |  |  |  |  |  |  |
| **C14** | M | 46 |  |  |  |  | RNA |  | X | X |  |  |  |  |  |  |  |
| **C15** | M | 39 |  |  |  |  | RNA |  | X |  |  |  |  |  |  |  |  |
| **C16** | M | 50 |  |  |  |  | BMMC |  |  |  |  |  |  |  |  |  | X |
| **C17** | F | 65 |  |  |  |  | BMMC |  |  |  |  |  |  |  |  |  | X |
| **C18** | F | 64 |  |  |  |  | BMMC |  |  |  |  |  |  |  |  |  | X |

ID (patients’ codes): AML (1–75), MPN (N1–N12), MDS (S1–S11), healthy donors (C1–C18); G (gender): F, female; M, male; A (age at diagnosis); F (FAB classification): 0, 1, 2, 3, 4, 5, 6, s, secondary AML, t, therapy-related AML; MUTATIONS (at diagnosis): NS, not studied; C-R (cytarabine-response, only applicable for technique 6): R, responder; NR, non-responder; M (sampling moment): DG, diagnosis; ACT, after cytarabine treatment; RES, resistance; TYPE (sample type): RNA, RNA from bone marrow; MET, methanol-fixed bone marrow smear; BMMC, bone marrow mononuclear cell; % (blast percentage); TECHNIQUE: 1, *SRRM2* expression by qPCR; 2, *SRSF12* expression by qPCR; 3, RNA sequencing; 4, phospho-proteomic studies; 5, paired IHC analysis (diagnosis *vs* resistance) for phospho-SR and SR proteins; 6, paired IHC analysis (diagnosis *vs* after cytarabine treatment) for phospho-SR proteins; 7, IHC analysis at diagnosis for phosphoSR proteins; 8, *ex vivo* evaluation of H3B-8800 plus venetoclax; 9, toxicity studies of H3B-8800 plus venetoclax.

**SUPPLEMENTAL FIGURES**

**Figure S1**. ***SRSF12* gene expression is altered in AML and other myeloid diseases. (A)** 2ΔCt values for *SRSF12* expression in bone marrow samples from patients with AML (n=16), MDS (n=11) and MPN (n=11) and healthy controls (n=14) at diagnosis, normalized to GUS expression.


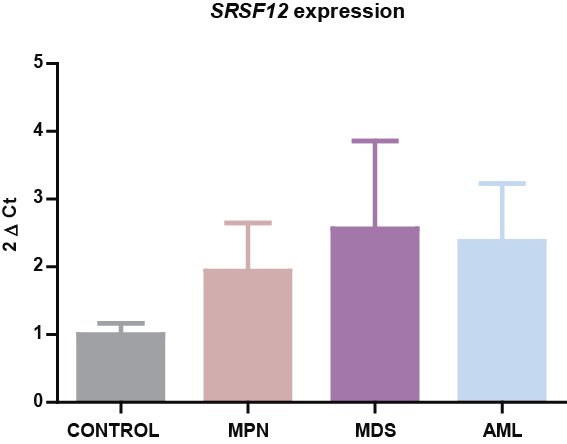


**Figure S2. RNA sequencing data analysis. (A)** Heatmap showing normalized and scaled expression of top 1000 most variable genes across samples. **(B)** Heatmap showing distances between samples. **(C)** Barplot representing library size for each sample.

**(D)** Principal component analysis (PCA) showing main sources of variability in the data (PC1 and PC2), representing samples by dots, and coloured by cytology measurement of blasts in each sample. **(E)** Heatmap showing normalized and scaled gene expression of 8 gene clusters, where genes were clustered based on partitioning around medoids (PAM) algorithm. **(F)** Number of differentially expressed genes (DEGs) up- and down- regulated in each group, with a 20% FDR.

**
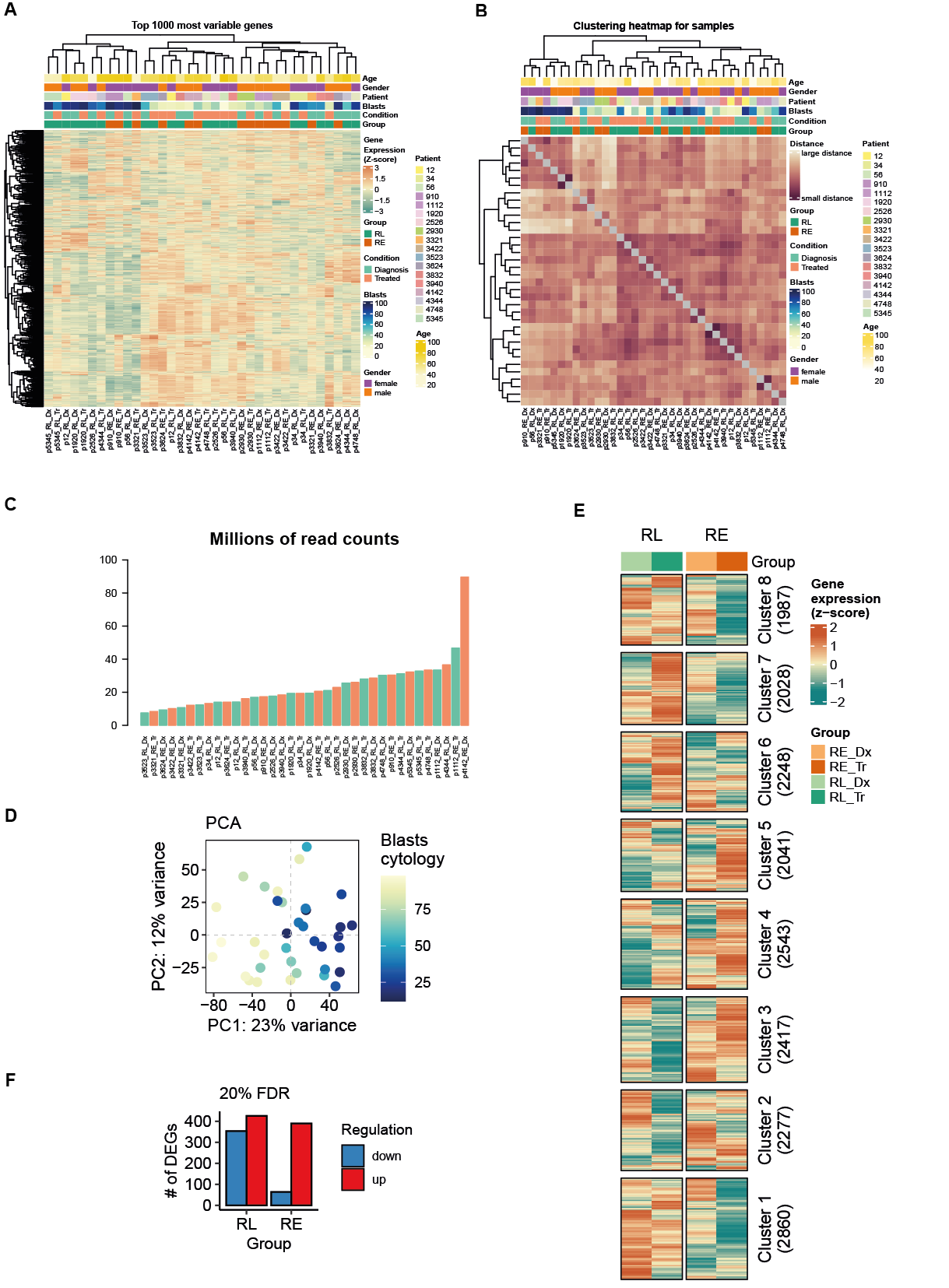
**

**Figure S3. Levels of phosphorylated SR proteins are elevated during cytarabine resistance. (A)** Bone marrow smear samples not included in the main text. Immunohistochemistry (IHC) staining of phospho-SR proteins in paired bone marrow smears at diagnosis and resistance in AML patients that responded to cytarabine and further relapsed or after cytarabine treatment in patients with AML that did not initially respond to treatment. **(B)** IHC and percentage of positive blasts, and nuclear and cytoplasmic staining of SR proteins in paired bone marrow smears at diagnosis and resistance in 4 patients with AML that responded to cytarabine and further relapsed.

**
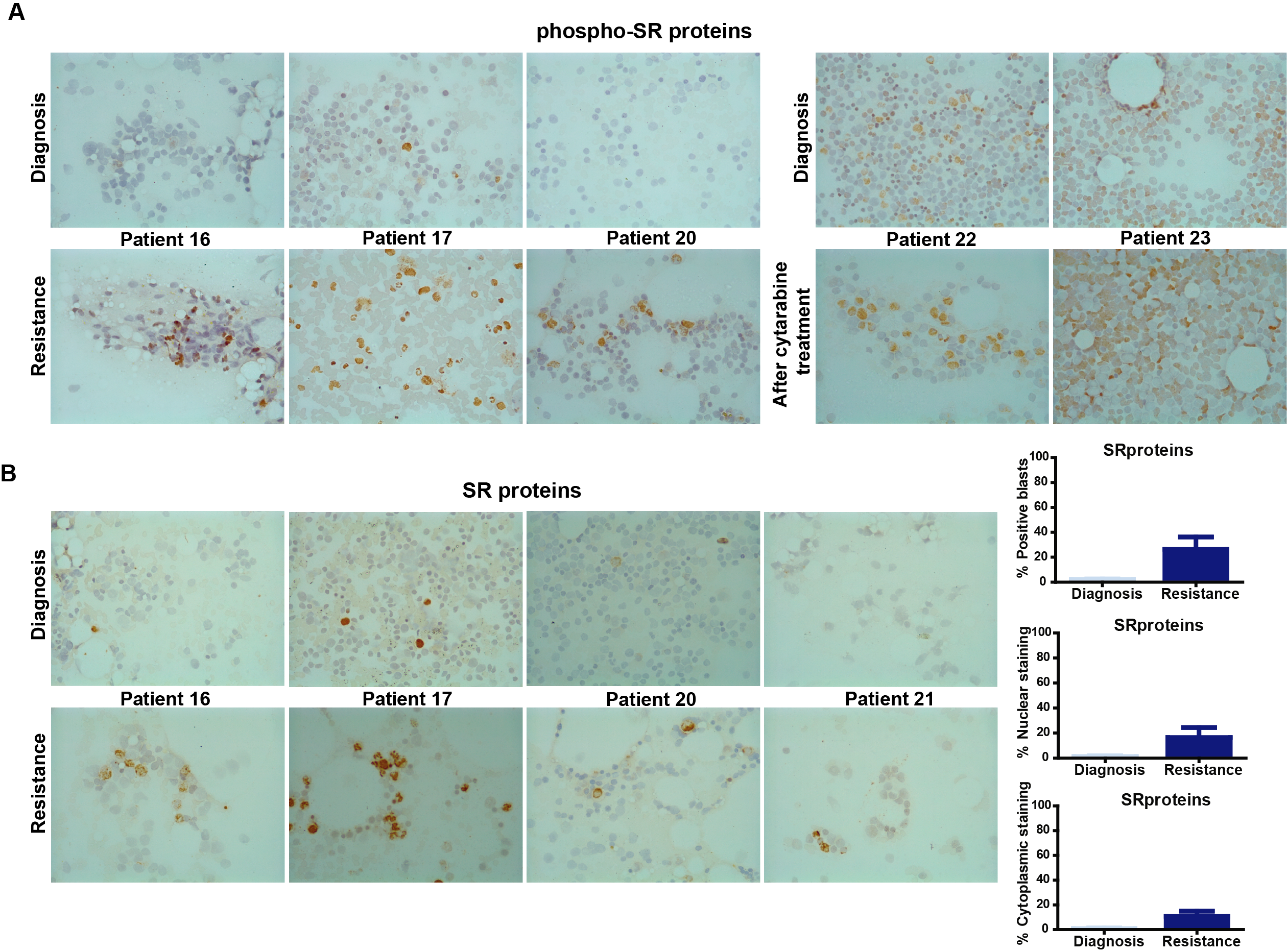
**

**Figure S4.** **Levels of phosphorylated SR proteins at diagnosis in responders and non-responders.** Bone marrow smears samples not included in the main text. IHC staining of phospho-SR proteins at diagnosis in 62 patients with AML classified as cytarabine responders or non-responders.

**
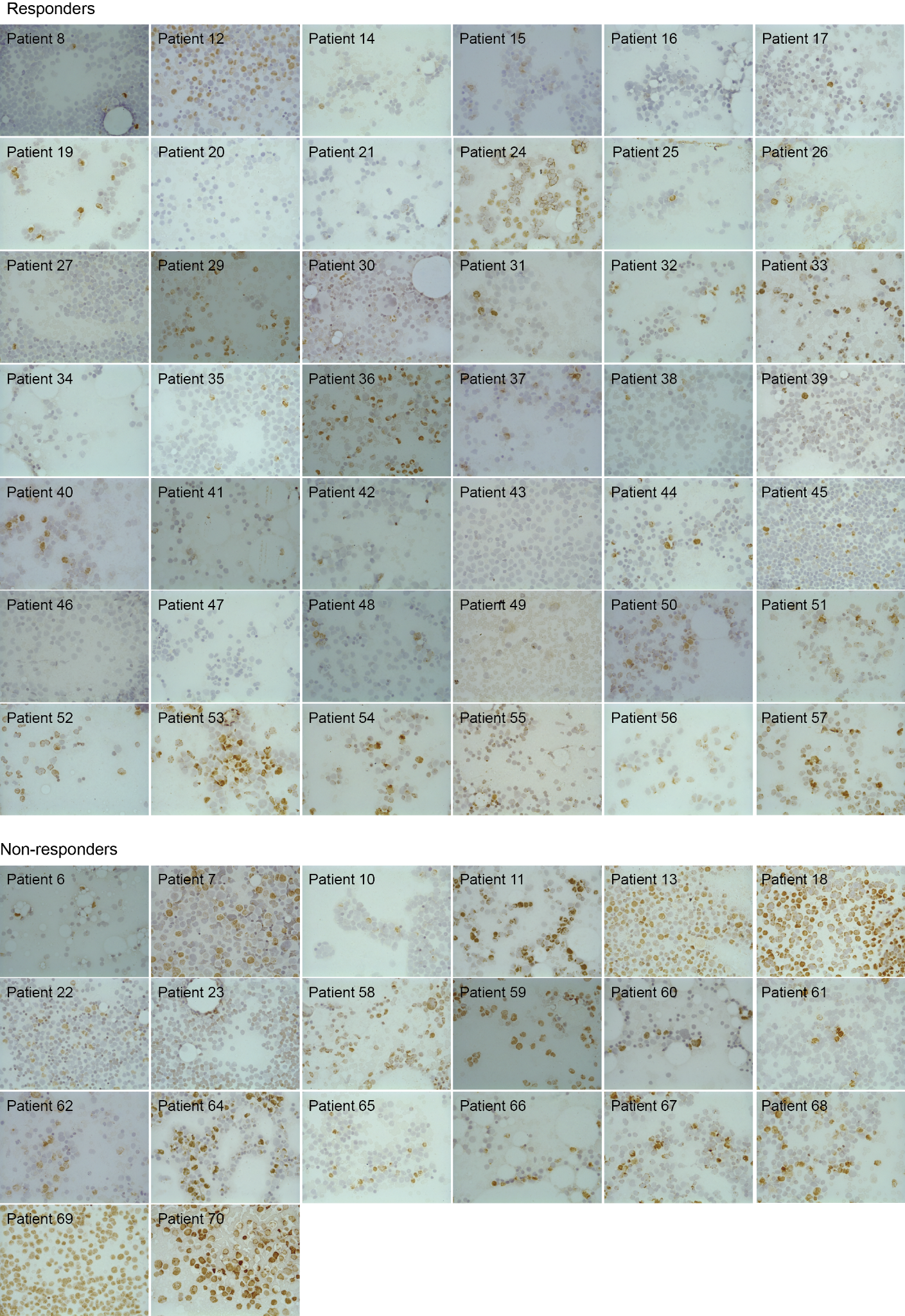
**

**Figure S5. Genetic alterations in cytarabine resistance. (A)** Mutational profile by functional categories of cytarabine responders (n = 38) and non-responders (n = 16) with AML whose samples were used for IHC studies. **(B)** Differential exon usage representations of the transcriptional profiles of paired samples (diagnosis *vs* resistance) from 25 patients with AML for the SR protein target genes *H2AFY, DPPIII, WAC, DEPDC5, Ki-67, MYLK,* and *S6K1*.

**
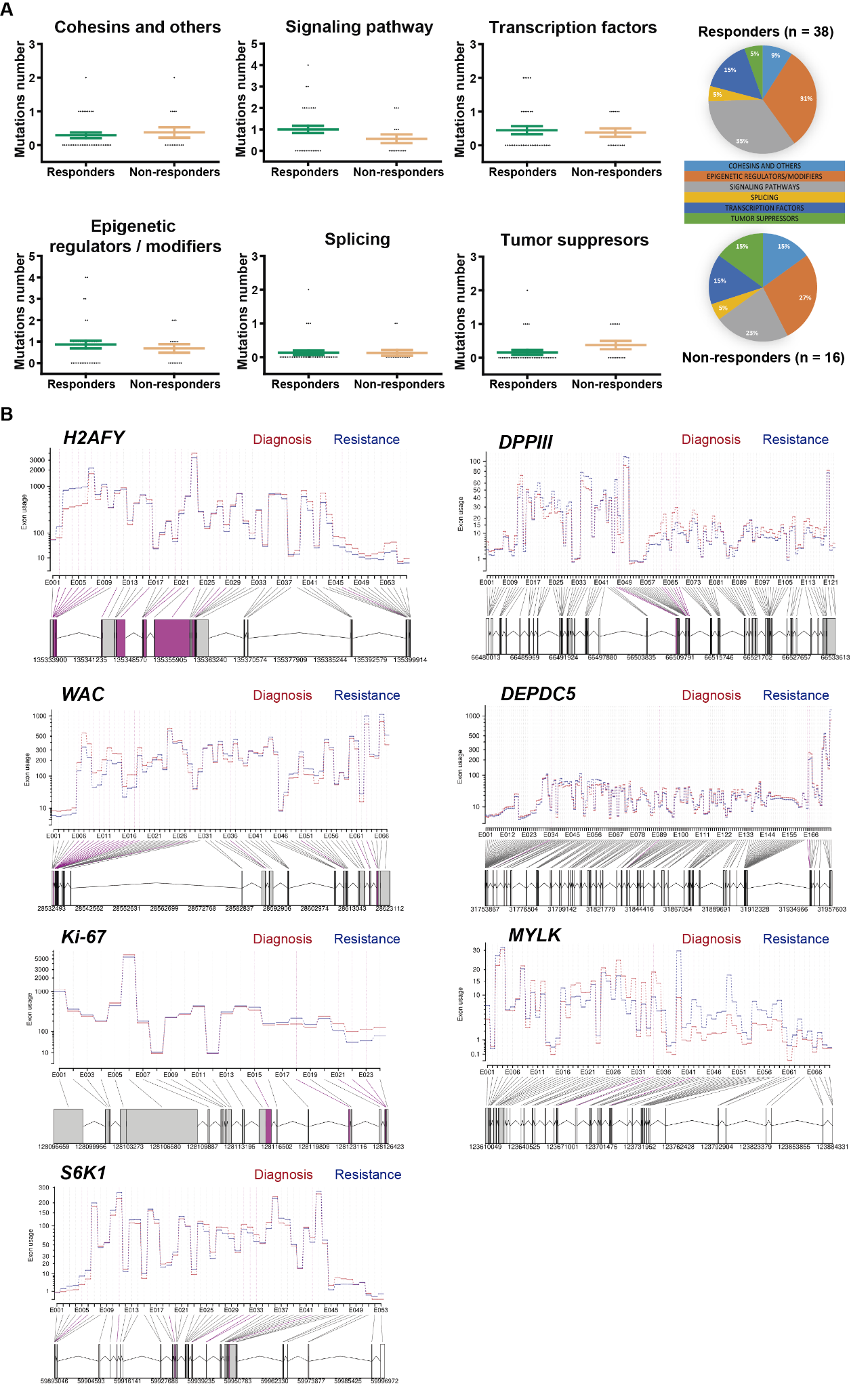
**

**Figure S6. *In vitro* evaluation of cytarabine and splicing inhibitors in cytarabine-sensitive and resistant AML cells. A)** Dose-response curves of OCI-AML3, SKM-1 and THP-1 cells for cytarabine and the splicing inhibitors H3B-8800, madrasin, SPHINX31 and SRPKIN-1. **B)** Dose-response curve of cytarabine-sensitive OCI-AML3 cells and the three selected OCI-AML3_R clones for cytarabine and the splicing inhibitors H3B-8800, madrasin, SPHINX31 and SRPKIN-1.


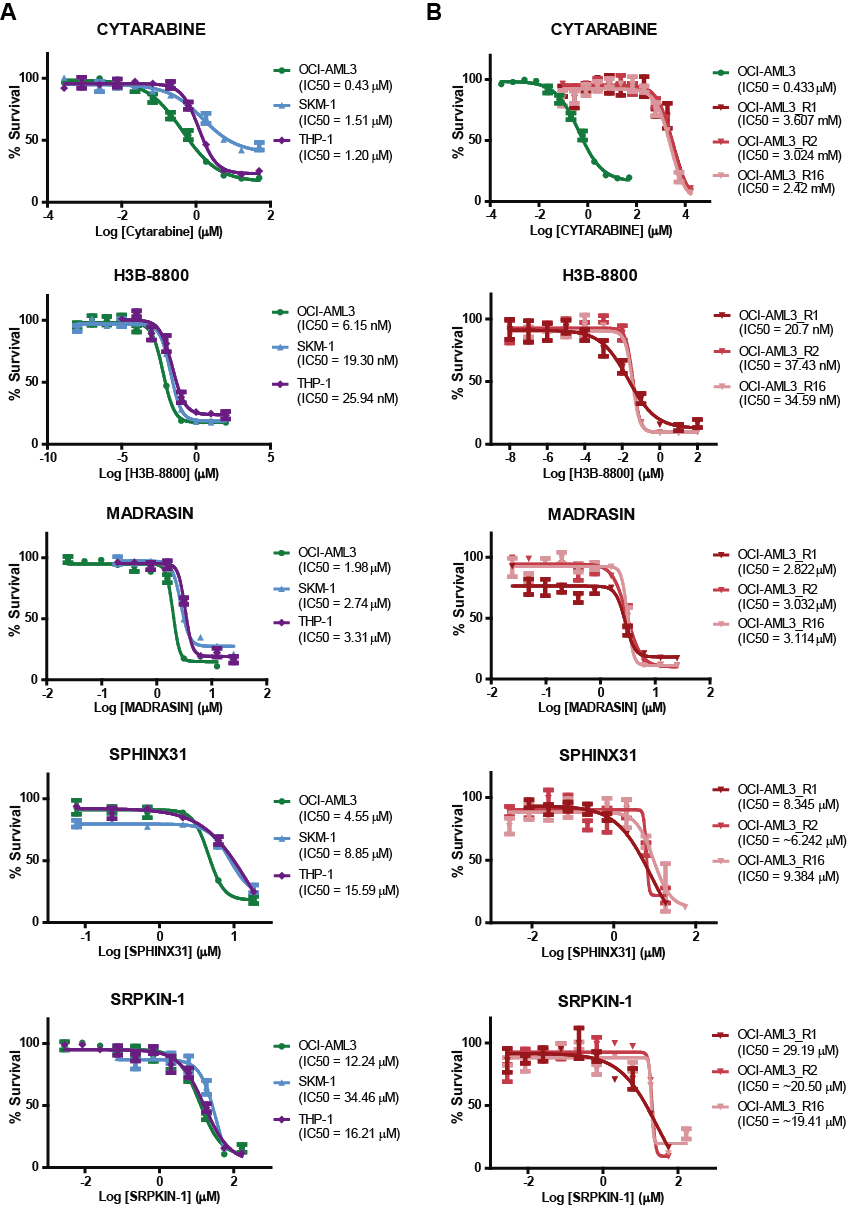


**Figure S7. Generation and validation of cytarabine-resistant OCI-AML3_R cell lines. (A)** OCI-AML3_R clone isolation scheme. **(B)** IHC and ratio of normalized staining of phospho-SR proteins in the cytarabine-sensitive OCI-AML3 and resistant cell models.

**
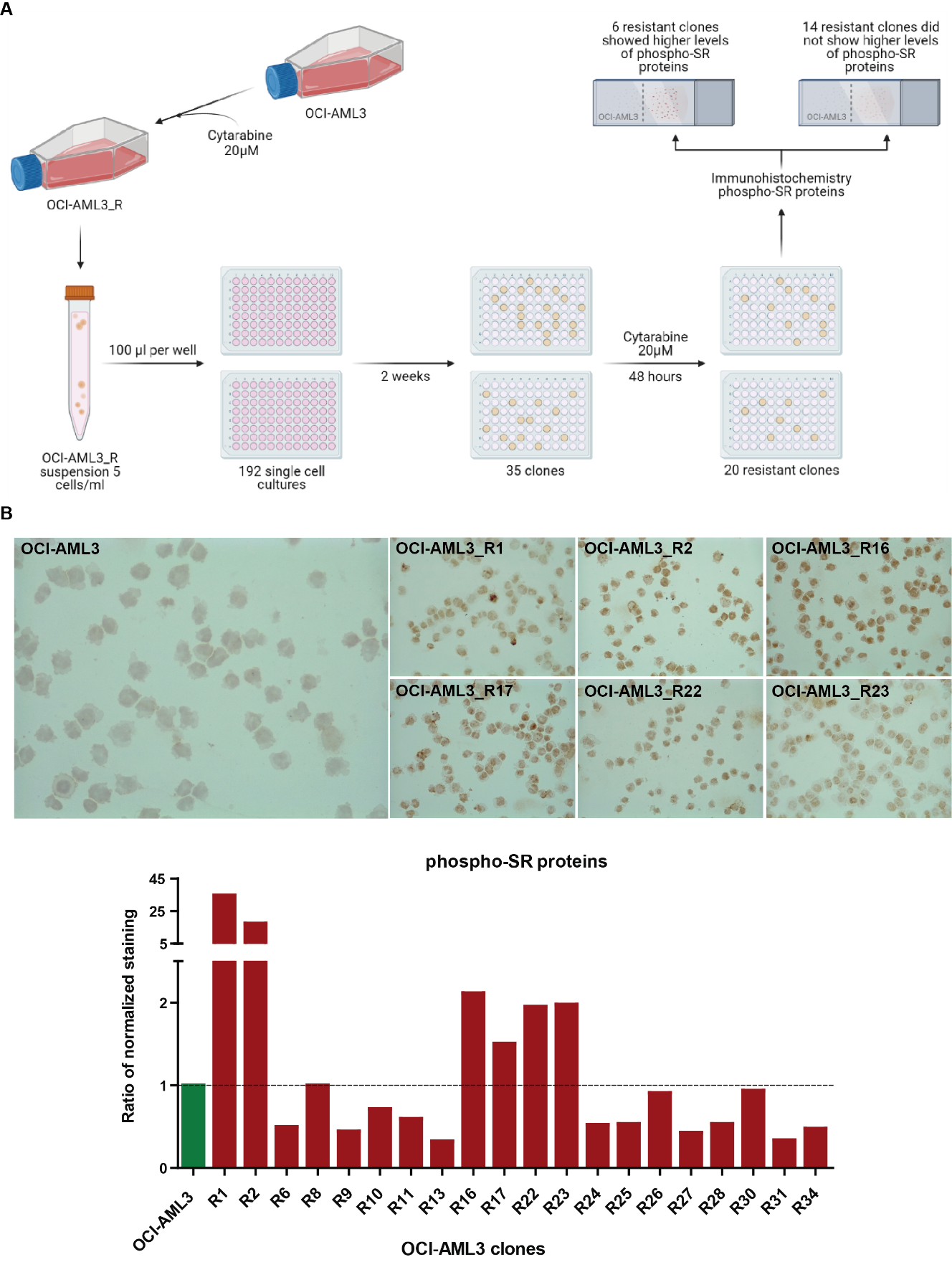
**

**Figure S8. *In vitro* evaluation of approved drugs for AML in cytarabine-sensitive and resistant cells.** Dose-response curves for azacitidine, decitabine, glasdegib, midostaurin, and venetoclax in OCI-AML3 and OCI-AML3_R1 cell lines.


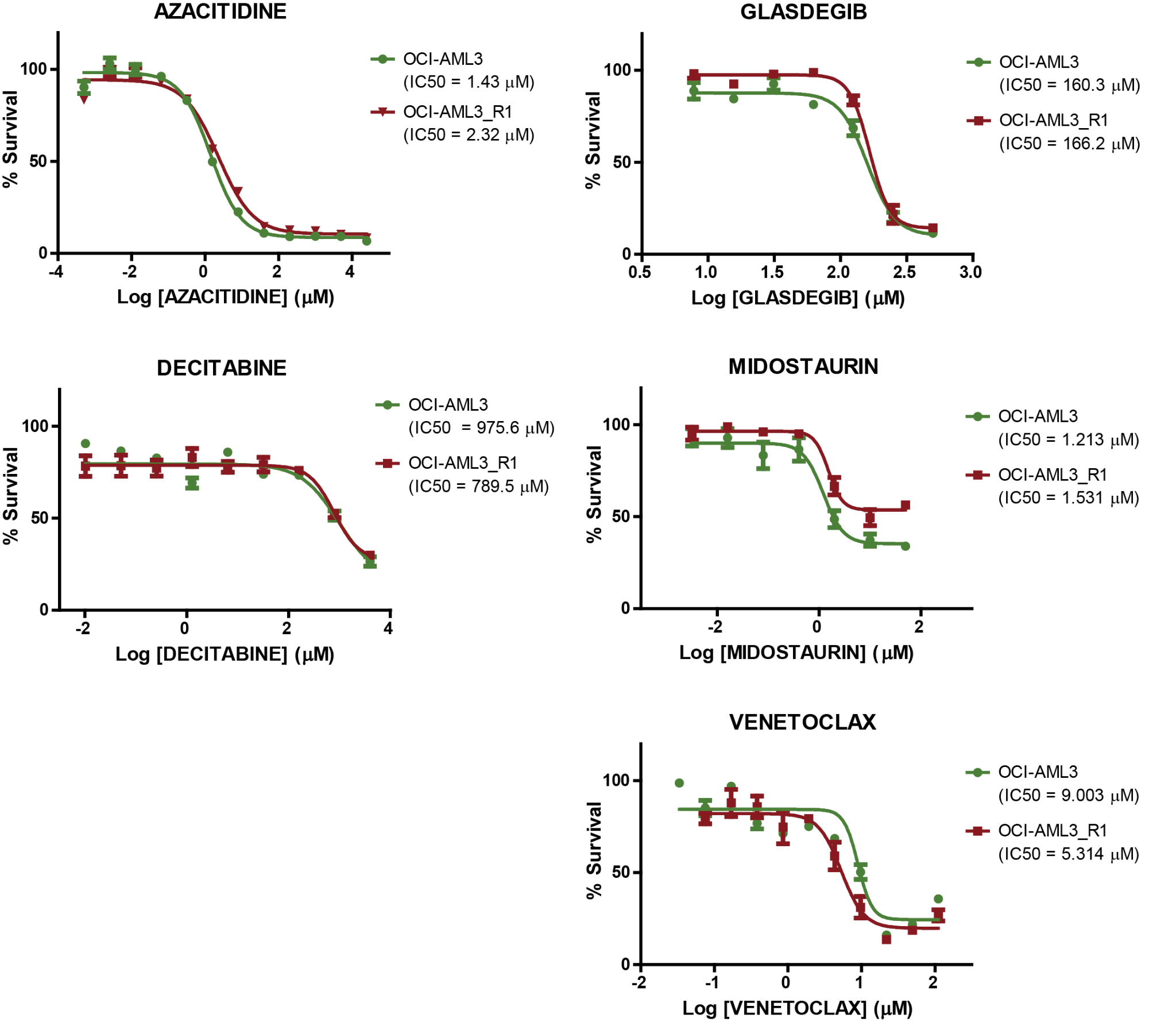


**Figure S9. Synergistic effect of H3B-8800 and other approved drugs in cytarabine-sensitive and resistant AML cells.** Normalized isobolograms for H3B-8800 in combination with azacitidine, decitabine, glasdegib or midostaurin in OCI-AML3 and OCI-AML3_R1 cell lines.

**
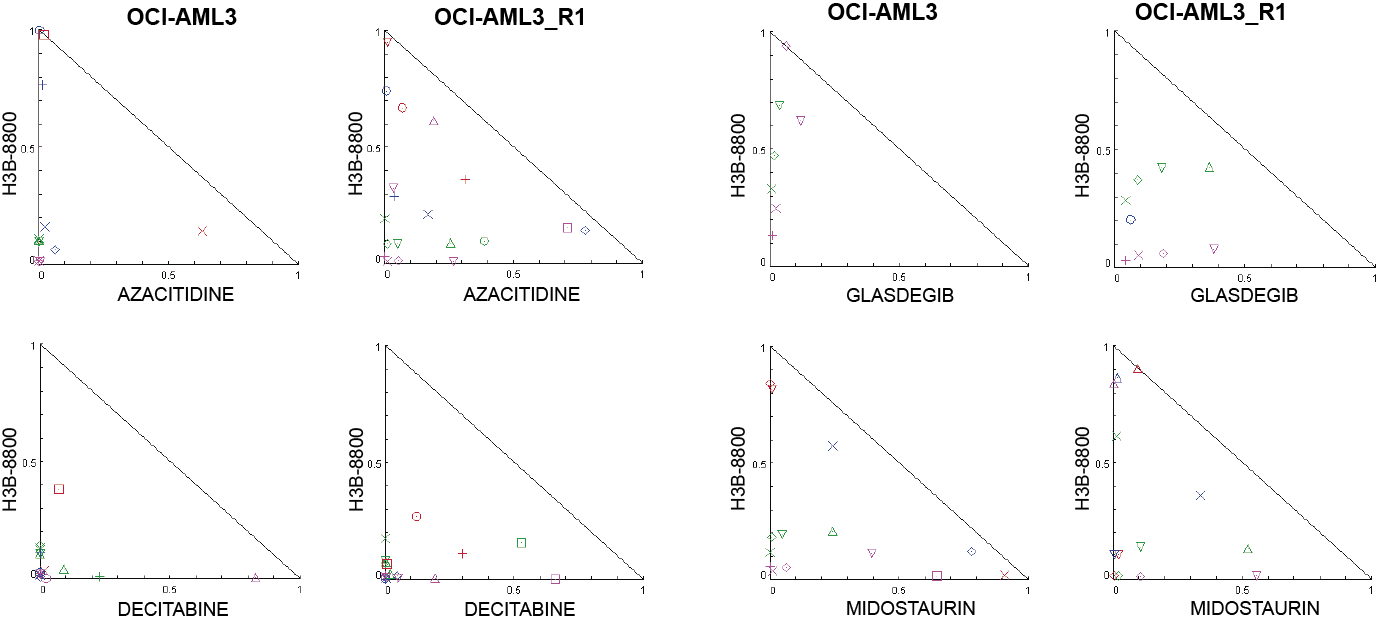
**
